# The genus *Alpioniscus* Racovitza, 1908 in Sardinia: taxonomy and natural history (Isopoda, Oniscidea, Trichoniscidae)

**DOI:** 10.3897/zookeys.801.24102

**Published:** 2018-12-03

**Authors:** Stefano Taiti, Roberto Argano, Paolo Marcia, Fabio Scarpa, Daria Sanna, Marco Casu

**Affiliations:** 1 Istituto di Ricerca sugli Ecosistemi Terrestri, Consiglio Nazionale delle Ricerche, Via Madonna del Piano 10, 50019 Sesto Fiorentino (Florence), Italy; 2 Museo di Storia Naturale dell’Università di Firenze, Sezione di Zoologia “La Specola”, Via Romana 17, 50125 Florence, Italy; 3 Dipartimento di Biologia e Biotecnologie “Charles Darwin”, Università degli Studi “La Sapienza”, Viale dell’Università 32, 00185 Rome, Italy; 4 Dipartimento di Medicina Veterinaria, Via Vienna 2, Università degli Studi di Sassari, 07100 Sassari, Italy; 5 Dipartimento di Scienze Biomediche, Viale San Pietro 43/C, Università degli Studi di Sassari, 07100 Sassari, Italy

**Keywords:** *
Alpioniscus
*, caves, Crustacea, new species, phylogeny, Sardinia

## Abstract

The genus *Alpioniscus* Racovitza, 1908 (Trichoniscidae) from Sardinia is revised. Three new cave-dwelling species are described: *A.onnisi* Taiti & Argano, **sp. n.**, *A.stochi* Taiti & Argano, **sp. n.**, and *A.sideralis* Taiti & Argano, **sp. n.**. The genus *Utopioniscus* Schmalfuss, 2005 is considered to be a junior synonym of *Alpioniscus*, after morphological and molecular analyses. *Alpioniscusfragilis* (Budde-Lund, 1909) and *A.kuehni* from Grotta del Bue Marino are illustrated. With the new species, the genus *Alpioniscus* in Sardinia comprises six species: two troglobionts (*A.fragilis* and *A.onnisi*), one endogean and troglobiont (*A.thanit* Taiti & Argano, 2009), and three stygobionts (*A.kuehni*, *A.stochi*, and *A.sideralis*). All the species occur in karstic areas in the central-eastern and south-eastern part of the island. A key to all the Sardinian species of *Alpioniscus* is provided.

## Introduction

At present, 92 species of terrestrial isopods are known from Sardinia (Taiti and Argano 2009, 2011), many of which strictly endemic. Several new species already identified during field investigations in the last years are waiting to be described. The aim of this study is to attempt a reconstruction of the biogeographic history of the genus *Alpioniscus* Racovitza, 1908 in Sardinia on the basis of new data.

The geographical range of the genus *Alpioniscus* is discontinuous. To date, the genus comprises 31 subterranean species in two subgenera (see Tabacaru 1966): the nominal subgenus with 14 species living in the caves of the Western Alps and the southern Balkans reaching Greece, and the subgenus Illyrionethes Verhoeff, 1927, with 17 species populating caves of Catalonia, Sardinia and the Dinaric Alps (Bedek and Taiti 2011, Bedek et al. 2017). In Sardinia two species are known in the subgenus Illyrionethes (Taiti and Argano 2009, 2011): *A.fragilis* (Budde-Lund, 1909), widely distributed in the karst caves of central-eastern and south-eastern areas of the island, and *A.thanit* Taiti & Argano, 2009, from endogean environments and some caves in the central-eastern area.

A large number of specimens have been recently collected from many new localities (mainly caves) on Sardinia, revealing a more complex taxonomic scenario. In the same part of Sardinia, Schmalfuss (2005) described a very interesting new species and genus of trichoniscids, the aquatic *Utopioniscuskuehni* Schmalfuss, 2005, occurring in two submarine caves. This species was considered among the most archaic forms of the family Trichoniscidae. New populations of this species have recently been examined from subterranean freshwaters in coastal and inland caves, and other stygobiotic species with intermediate characters between *Alpioniscus* and *Utopioniscus* have also been identified. In this paper three new species of *Alpioniscus* are described and the synonymy between *Alpioniscus* and *Utopioniscus* proposed, on the basis of both morphological and molecular analyses. Analyses performed on both morphological and molecular techniques allow an exhaustive integrative taxonomic approach, which has been effectively used in several case studies on small-sized faunal taxa (see e.g., Casu et al. 2011, 2014, Scarpa et al. 2016, 2017a, b).

## Materials and methods

### The study area

Sardinia is the second largest island of the Mediterranean (24,090 km^2^) with a complex geology. Karsts cover 9% of the total surface and are divided in 219 distinct areas of different ages, ranging from sea level up to 1,500 m altitude. These areas are separated from each other by non-karstic rocks, so they evolve and behave independently to one another (De Waele 2003, 2009). *Alpioniscus* species in Sardinia are limited to the central-eastern and south-eastern main karst groups (Gulf of Orosei, Supramonte, Tacchi, Quirra, and Sarrabus).

### Collectors of materials

The specimens examined were collected by some of the authors, several biospeleologists, and the astronauts from Europe, USA, Russia, Canada, Japan, and China, participating in the ESA CAVES training courses.

### Morphological analysis

All material collected for morphological analysis was stored in 75% ethanol. The species were illustrated with the aid of a *camera lucida* mounted on Wild M5 and M20 microscopes. Figures were digitally drawn following the methods described in Montesanto (2015, 2016). The World Geodetic System 1984 (WGS84) was the datum used for all geographic coordinates.

### Molecular analysis

Several specimens of each Sardinian species from the type localities have been tested for molecular analysis (Table 1), including one specimen of *Utopioniscuskuehni* studied by Schmalfuss (2005). Moreover, in order to test the assignment of the Sardinian species of *Alpioniscus* to the subgenus Illyrionethes we included also specimens of *Alpioniscusstrasseri* (Verhoeff, 1927), type species of the subgenus Illyrionethes from Friuli Venezia Giulia (Italy), and of *A.feneriensis* (Parona, 1880), type species of the subgenus Alpioniscus Racovitza, 1908, from Piedmont (Italy). A specimen of *Androniscusdentiger* Verhoeff, 1908 (fam. Trichoniscidae) from Tuscany (Italy) was used as outgroup.

Molecular analyses have been performed using the COI gene (Cytochrome c Oxidase subunit I) with either the universal COI primers by Folmer et al. (1994) or new specific primers designed by the authors (H: grgatgaycaratytayaatgt, L: ctaggrtcaaaaaarcawgtgtt). DNA extraction and PCR have been performed following Sanna et al. (2014). Annealing temperature was set at 44° C for both of primers pairs; positive and negative controls were also used for PCR.

PCR products were purified by ExoSAP-IT (USB Corporation) and sequenced using an external sequencing core service (Macrogen Inc., Europe). The sequencing runs were performed both for forward and reverse strands. Sequences were aligned using Clustal W (Thompson et al. 1994), implemented in BioEdit 7.0.5.2 software (Hall 1999). The best probabilistic model of sequence evolution was determined after evaluation by jModeltest 2.1.1 (Posada 2008), with a maximum likelihood optimized search, using the Akaike Information Criterion (AIC) and the Bayesian Information Criterion (BIC). The model TPM2uf + G has been chosen as the best fitting both AIC and BIC. Phylogenetic relationships were investigated using the Bayesian Inference (BI) and the Maximum Likelihood (ML) methods. BI was carried out using the software MrBayes 3.2.2 (Ronquist et al. 2012), setting as model parameters: N_ST_ = 3, rates = gamma, ngammacat = 4. Two independent runs each consisting of four Metropolis-coupled MCMC chains (one cold and three heated chains) were run simultaneously for 5,000,000 generations, sampling trees every 1,000 generations. The first 25% of sampled trees were discarded. Run was executed by means of the Cipres Phylogenetic Portal (Miller et al. 2010). Convergence of chains was checked following the procedures described by Ronquist et al. (2012) and Gelman and Rubin (1992). ML analysis were conducted using the software RAxMLGUI version 1.3 (Silvestro and Michalak 2011) setting the default setting for the “ML + thorough bootstrap” analysis option. Analysis was carried out with 100 runs and 1,000 bootstrapping replicates. Consensus trees were visualized by means of the FigTree 1.4.0 software (http://tree.bio.ed.ac.uk/software/figtree/).

**Table 1.** Specimens used for molecular analysis.

**Species**	**Collection data**	**GenBank number**
*Alpioniscusfeneriensis* (Parona, 1880)	Piedmont: Buco della Bondaccia, c.n. 2517 Pi/VC, Borgosesia, Monte Fenera, 30.IX.2000, leg. F. Stoch, T. Pascutto and S. Bugalla	MH092992
*Alpioniscusstrasseri* (Verhoeff, 1927)	Friuli Venezia Giulia: Grotta del Bosco dei Pini, c.n.16 VG/TS, Basovizza, 2.VI. 2011, leg. F. Gasparo and F. Stoch	MH092986
*Alpioniscusfragilis* (Budde-Lund, 1909)	Sardinia: Grotta del Bue Marino, 25.IV.2012, leg. S. Taiti, P. Dore and S. Dessena	MH092990
*Alpioniscuskuehni* (Schmalfuss, 2005)	Sardinia: Grotta del Bel Torrente (Paratype), 22.VII, 2004, leg. A. Oertel	MH092988
*Alpioniscusthanit* Taiti & Argano, 2009	Sardinia: Cala Fuili, 25.IV.2008, leg. R. Argano and S. Taiti	MH092993
*Alpioniscusonnisi* sp. n.	Sardinia: Grotta Giuanniccu Mene, 20.IV.2012, leg. C. Onnis, S. Taiti and R. Argano	MH092987
*Alpioniscusstochi* sp. n.	Sardinia: Grotta Su Palu, 1.V.2009, leg. F. Stoch and G. Tomasin	MH092991
*Alpioniscussideralis* sp. n.	Sardinia: Grotta Su Bentu, 11-14.IX.2012, leg. P. Marcia and ESA astronauts	MH092989
*Androniscusdentiger* Verhoeff, 1908 (outgroup)	Tuscany: Tana di Magnano, 162 To/LU, Villa Collemandina, 11.VI.2012, leg. S. Taiti	MH092985

### Abbreviations

**c.n.** Cadastral number;

**ESA CAVES** European Space Agency, Cooperative Adventure for Valuing and Exercising human behaviour and performance Skills;

**MZUF** Museo di Storia Naturale dell’Università di Firenze, Sezione di Zoologia “La Specola”, Florence, Italy;

**n.c.n.** No cadastral number;

**SMNS** Staatliches Museum für Naturkunde, Stuttgart, Germany.

## Taxonomic results

### Family Trichoniscidae Sars, 1899

#### Genus *Alpioniscus* Racovitza, 1908

##### Alpioniscus fragilis

Taxon classificationAnimaliaIsopodaTrichoniscidae

(Budde-Lund, 1909)

**Figure 1.**
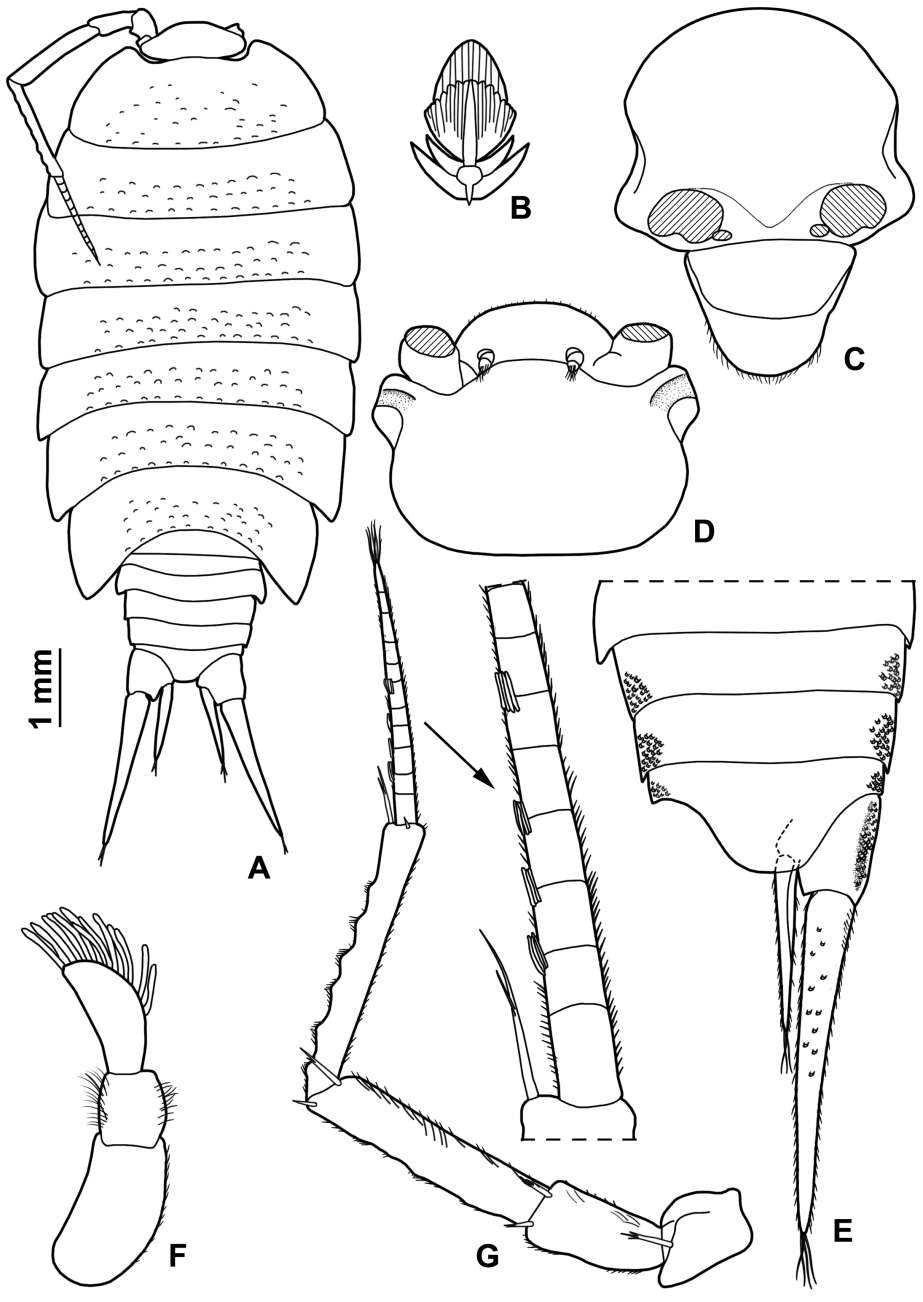
*Alpioniscusfragilis* (Budde-Lund, 1909) from Grotta del Bue Marino, ♂: **A** adult specimen, dorsal **B** dorsal scale-seta **C** cephalon, frontal **D** cephalon, dorsal **E** pleonites 3-5, telson and right uropod **F** antennula **G** antenna.

**Figure 2.**
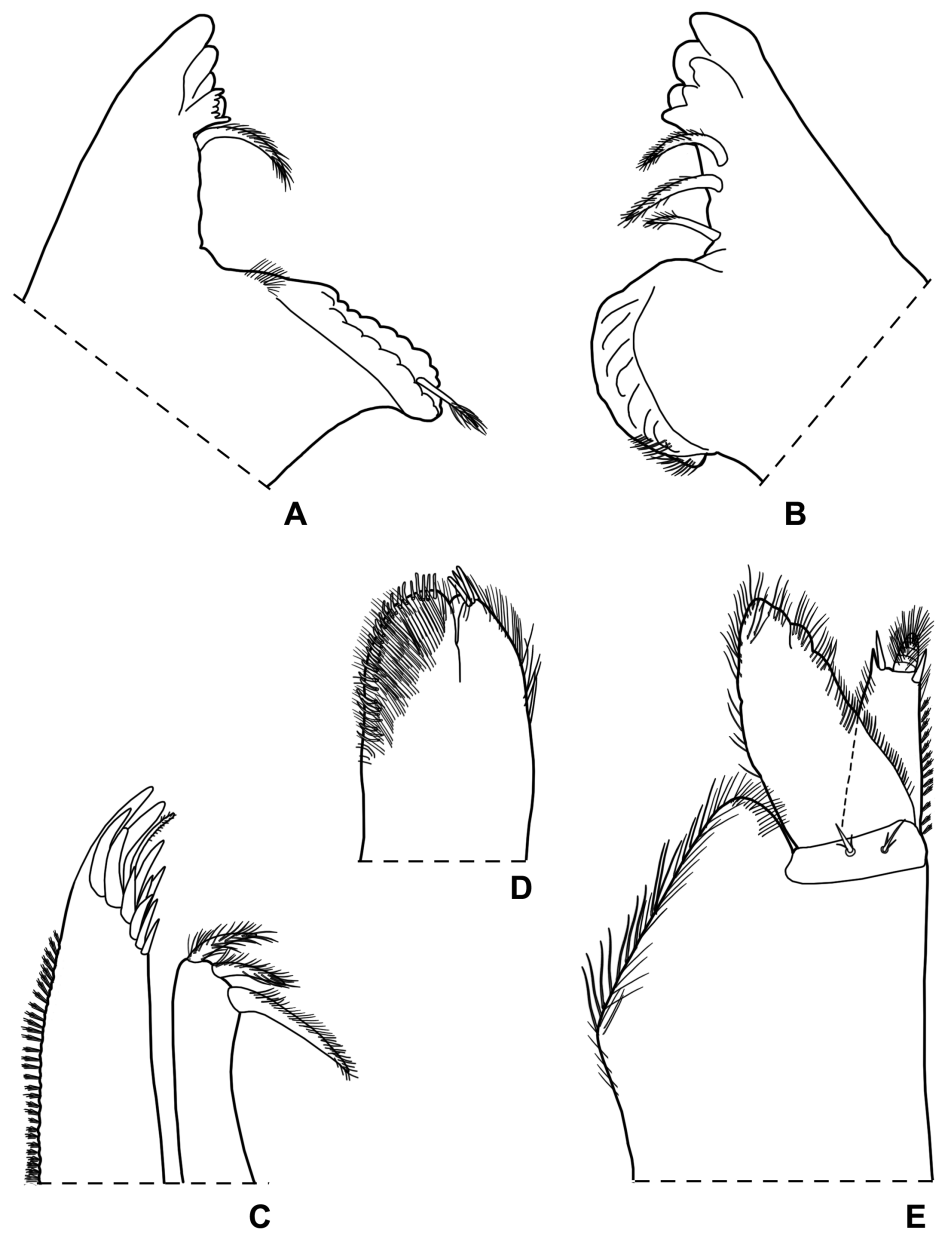
*Alpioniscusfragilis* (Budde-Lund, 1909) from Grotta del Bue Marino, ♂: **A** right mandible **B** left mandible **C** maxillula **D** maxilla **E** maxilliped.

**Figure 3.**
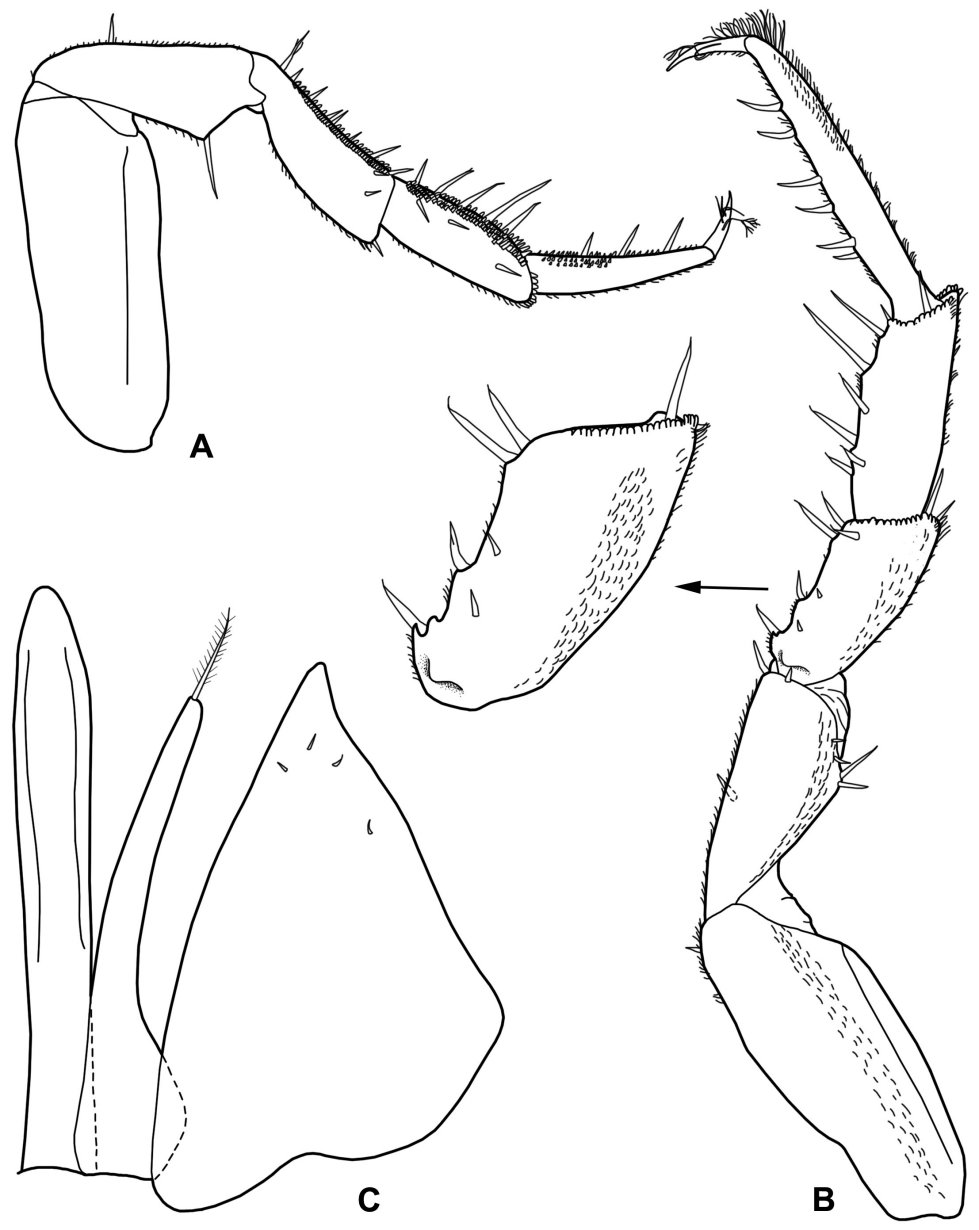
*Alpioniscusfragilis* (Budde-Lund, 1909) from Grotta del Bue Marino, ♂: **A** pereopod 1 **B** pereopod 7 **C** genital papilla and pleopod 1.

**Figure 4.**
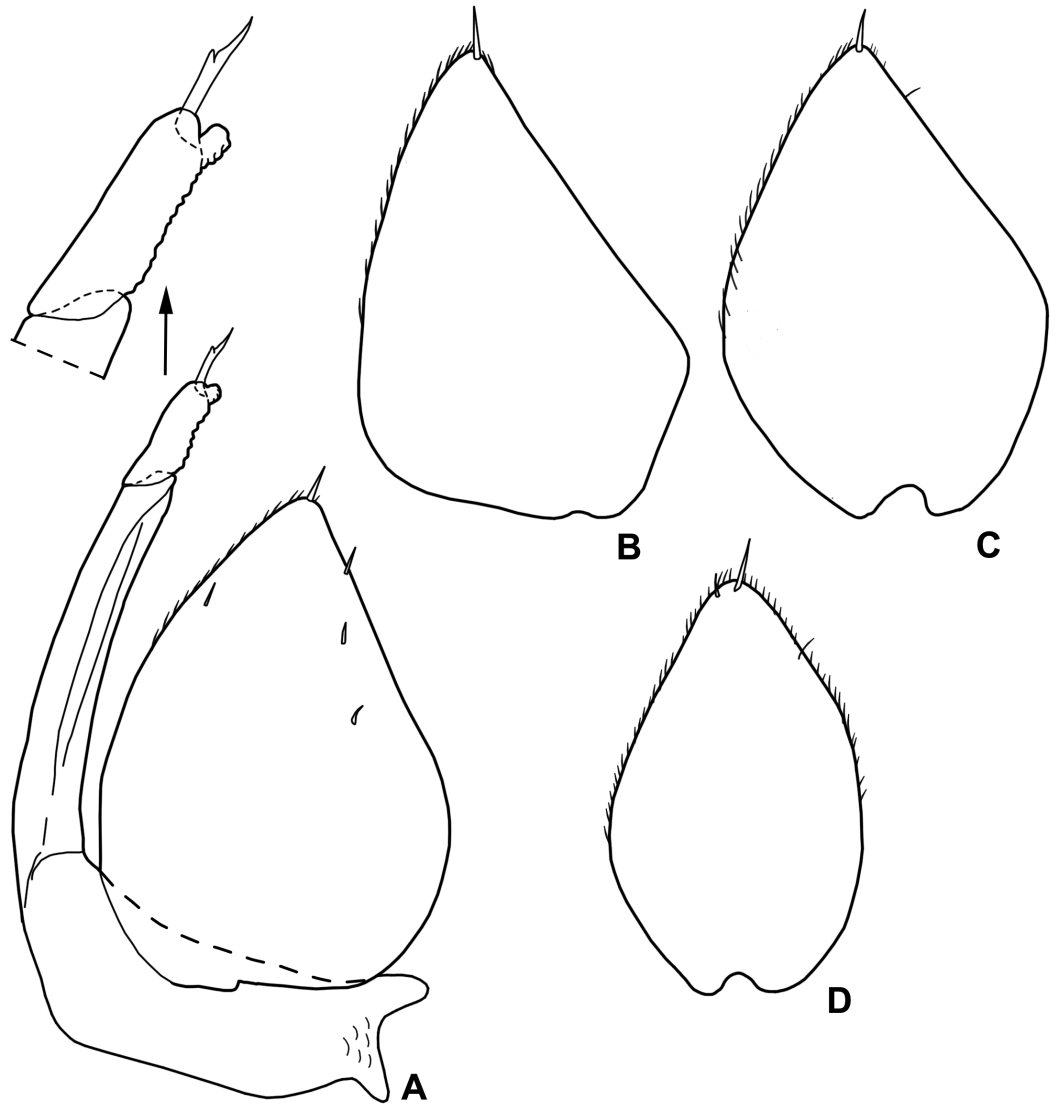
*Alpioniscusfragilis* (Budde-Lund, 1909) from Grotta del Bue Marino, ♂: **A** pleopod 2 **B** pleopod 3 exopod **C** pleopod 4 exopod **D** pleopod 5 exopod.

Figs 1 , 2 , 3 , 4 , 18 , 19

Alpioniscus fragilis ; Taiti and Argano 2011: 166 (for previous records and references; nec Grotta del Caprone Tyson, p. 167).

###### Material examined.

**Prov. Nuoro**: 5 ♂♂, 16 ♀♀, 1 juv. (MZUF 9770), Grotta del Bue Marino, c.n. 12 Sa/NU, 40°14'55.72"N, 9°37'24.80"E, Cala Gonone, Dorgali, on cave walls, 25.IV.2012, leg. S. Taiti, P. Dore and S. Dessena; 1 ♂, 4 ♀♀ (MZUF 9826), same locality, date and collectors, under submerged stones; 1 ♀ (MZUF 9774), Grotta Pisanu or Gurennoro, c.n. 215 Sa/NU, 40°17'56.40"N, 9°33'05.30"E, 142 m, Gurennoro, Dorgali, 17.III.2014, leg. P. Magrini; 2 ♂♂, 1 ♀, 15 juvs (MZUF 9777), Grotta Elighes Artas, c.n. 907 Sa/NU, 40°14'20.3"N, 9°28'49.8"E, 360 m, Oliena, 25.IV.2013, leg. P. Marcia and S. Taiti; 1 ♂, 2 juvs (MZUF 9778), same locality, 15.I.2012, leg. P. Marcia; 1 ♂, 2 ♀♀, 3 juvs (MZUF 9779), same locality, 25.XII.2012, leg. G. Mulas; 2 ♂♂, 4 ♀♀, 2 juvs (MZUF 9780), Grotta Su Bentu, c.n. 105 Sa/NU, 40°15'18.23"N, 9°29'6.52"E, Lanaittu, Oliena, 3.XII.2011, leg. P. Marcia; 5 ♂♂, 3 ♀♀ (MZUF 9781), same locality, 6.I.2013, leg. P. Marcia; 2 ♀♀, 1 juv. (MZUF 9782), same locality 11-14.IX.2012, leg. P. Marcia and Astronauts; 1 ♂, 2 ♀♀ (MZUF 9783), same locality, 4.XII.2011, leg. P. Marcia; 4 ♂♂, 6 ♀♀, 9 juvs (MZUF 9784), Grotta S’Istampu de Sas Ballas, c.n. 106 Sa/NU, 40°15'20.82"N, 9°29'13.72"E, Oliena, 30.XII.2012, leg. P. Marcia; 4 ♀♀ (MZUF 9785), Grotta sa Seneppida, n.c.n., sa Seneppida, Orgosolo, 13.I.2013, leg. E. Dallocchio; 4 ♂♂, 8 ♀♀, 6 juvs (MZUF 9823), Voragine di Tiscali, c.n. 88 Sa/NU, 40°14'12.23"N, 9°29'6.52"E, Oliena, 23.X.2011, leg. P. Marcia. **Prov. Ogliastra**: 2 ♀♀ (MZUF 9772), Grotta Lovettecannas, c.n. 2642 Sa/OG, 40°08'33.72"N, 9°34'35.35"E, Baunei, 1.IV.2013, leg. P. Marcia; 1 ♂, 1 ♀ (MZUF 9773), Grotta di Baccherutta, c.n. 1008 Sa/OG, 40°04'5.22"N, 9°37'34.54"E, Baunei, 14.III.2009, leg. C. Onnis and N. Ibba; 1 ♂ (MZUF 9786), Grotta Piggios de Jana, n.c.n., Tauledda, Codula del Flumineddu, Urzulei, 28.X.2012, leg. C. Corongiu; 1 ♂ (MZUF 9787), Grotta Sa rutta e Mannaresuru, c.n. 2267 Sa/OG, 40°07'44.64"N, 9°26'49.99"E, Urzulei, 15.VII.2012, leg. P. Marcia; 4 ♂♂, 14 ♀♀ (MZUF 9788), same locality, 7.X.2012, leg. P. Marcia; many ♂♂ and ♀♀ (MZUF 9789), Grotta Sa Rutta ‘e s’Edera, c.n. 588 Sa/OG, 40°05'51.3"N, 9°27'22.5"E, 950 m, Fennau, Urzulei, 23.IV.2012, leg. R. Argano and S. Taiti; 1 ♂, 6 ♀♀, 5 juvs (MZUF 9790), Grotta Su Palu, c.n. 1988 Sa/OG, 40°10'38.23"N, 9°33'50.53"E, 185 m, Codula Ilune, Urzulei, 8.XII.2012, leg. P. Marcia; 1 ♀ (MZUF 9775), Grotta Su Molente, c.n. 966 Sa/OG, 40°13'00.90"N, 9°36'10.95"E, Codula Ilune, Dorgali, 9.XI.2013, leg. M. Marrosu; 1 ♀ (MZUF 9807), same locality, 10.XI.2013, leg. E. Seddone; 4 ♂♂, 7 ♀♀ (MZUF 9771), Grotta Sos Cicinderos, n.c.n., Baunei, 7.VII.2013, leg. C. Onnis and M. Papacoda; 1 ♀ (MZUF 9791), Voragine Tesulali, c.n. 2681 Sa/OG, 40°07'42.14"N, 9°34'50.82"E, Baunei, 26.II.2012, leg. C. Onnis; 7 ♂♂, 19 ♀♀ (MZUF 9792), same locality, 10.II.2013, leg. C. Onnis; 1 ♂, 1 juv. (MZUF 9793), Grotta Su Tufu de Mangalistru, c.n. 422 Sa/OG, 40°06'17.28"N, 9°39'09.03"E, Baunei, 25.III.2012, leg. C. Corongiu; 1 ♀ (MZUF 9794), Grutta ’e S’Arena, c.n. 673 Sa/OG, 39°51'04.29"N, 9°27'44.43"E, Taquisara, 22.V.2013, leg. C. Onnis and P. Marcia; 1 ♂, 1 ♀ (MZUF 9798), same locality, 15.IX.2013, leg. C. Onnis and J. Costantino; 4 ♂♂, 5 ♀♀ (MZUF 9795), Grotta Istirzili, c.n. 50 Sa/NU, 40°04'49.50"N, 9°37'13.40"E, II..2013, leg. C. Onnis; 4 ♂♂, 7 ♀♀, 2 juvs (MZUF 9796), same locality, 12.V.2013, leg. C. Onnis; 3 ♂♂, 8 ♀♀ (MZUF 9797), Grotta S’erriu Mortu, n.c.n., Punta Giradili, Baunei, 7.VII.2013, leg. C. Onnis and M. Papacoda; 1 ♀ (MZUF 9799), Sa Grutta de su Coloru, c.n. 670 Sa/OG, 39°50'54.20"N, 9°27'34.68"E, Gairo Taquisara, 15.IX.2013, leg. C. Onnis and J. Costantino; 1 ♀ (MZUF 9801), same locality, 21.VIII.2013, leg. C. Onnis; 2 ♂♂, 21 ♀♀ (MZUF 9800), Lequarci or Lecorci Falls, Santa Barbara, Ulassai, 39°47'29.2"N, 9°27'11.6"E (WGS84), 560 m, under big stones outside cave, 28.III.2016, leg. R. Argano and S. Taiti. **Prov. Cagliari**: 1 ♂, 1 ♀, 2 juvs (MZUF 9824), Grotta Gospuru, c.n. 148 Sa/CA, 39°31'48.31"N, 9°26'13.21"E, 100 m, Baccu Gospuru, Armungia, IV.2012, leg. C. Onnis; 1 ♀, 2 juvs (MZUF 9776), Grotta Su Pittiolu de Gospuru, c.n. 1865 Sa/CA, 39°31'59.65"N, 9°26'04.41"E, 125 m, Baccu Gospuru, Armungia, 5.X.2008, leg. C. Onnis and N. Ibba; 2 ♂♂, 2 ♀♀ (MZUF 9825), same locality, 6.III.2011, leg. P. Marcia; 8 ♂♂, 15 ♀♀, 7 juv. (MZUF 9802), Grotta Su Fummu, n.c.n., San Nicolò Gerrei, 1.V.2012, leg. C. Onnis; 1 ♂, 12 ♀♀ (MZUF 9803), same locality, 17.III.2013, leg. C. Onnis; 2 ♂♂, 5 ♀♀ (MZUF 9804), Risorgenza Sa Gisterra, n.c.n., 39°30'08.4"N, 9°19'05.2"E, San Nicolò Gerrei, IX.2012, leg. C. Onnis; 2 ♀♀ (MZUF 9805), same locality, 20.IV.2013, leg. C. Onnis, R. Argano and S. Taiti; 3 ♂♂, 9 ♀♀ (MZUF 9806), Sa Rutt’e Scusi, c.n. 602 Sa/CA, 39°29'42.20"N, 9°24'18.00"E, Villasalto, V.2013, leg. C. Onnis.

###### Redescription.

Maximum length: ♂, 9 mm; ♀, 14 mm. Colourless body, pleon narrower than pereon (Fig. 1A). Dorsal surface distinctly granulated with ovoid scale-setae as in Fig. 1B. Many gland pores on lateral margins of pleonites 4 and 5, telson, lateral surface of uropodal protopods, and some scattered pores on dorsal surface of uropodal exopods (Fig. 1E). Cephalon (Fig. 1C, D) with suprantennal line V-shaped with rounded middle part; antennal lobes quadrangular, obliquely directed outwards with concave dorsal surface. Eyes absent. Posterior margin of pereonite 1 and 2 straight, and of pereonites 3–7 progressively more concave (Fig. 1A). Pleonites 3–5 with very short posterior points (Fig. 1A, E). Telson (Fig. 1E) approx. twice as wide as long; distal part with concave sides and very broadly rounded apex. Antennula (Fig. 1F) with second article distinctly shorter than first and third; third article distally enlarged and bearing 12–13 apical aesthetascs. Antenna (Fig. 1G) with fifth article as long as flagellum; flagellum of 10–13 articles with four groups of aesthetascs. Mandibles with one free penicil and one short molar penicil in the right (Fig. 2A) and three free penicils in the left (Fig. 2B). Maxillula (Fig. 2C) outer branch with 4 + 7 teeth, apically entire, and one slender setose stalk; inner branch with three long penicils. Maxilla (Fig. 2D) with setose and bilobate apex, inner lobe smaller. Maxilliped (Fig. 2E) basis with outer margin distally oblique and setose; palp stout, apically with tuft of setae and three tufts of setae on medial margin, basal article with two setae; endite narrow, with two apical stout setae and a large penicil. Pereopods with setose dactylar seta distally bifid (Fig. 3A), pereopod 7 with water conducting system on basis, ischium and merus. Uropod (Fig. 1E) with endopod distinctly shorter than exopod, endopod inserted proximally to exopod.

*Male*. Pereopod 1–4 (Fig. 3A) with carpus and merus bearing numerous short scales on sternal margin. Pereopod 7 (Fig. 3B) ischium with straight sternal margin covered with short setae; merus with three lobes proximally. Genital papilla (Fig. 3C) with a rounded tip. Pleopod 1 (Fig. 3C) exopod triangular with acute apex; endopod enlarged at base, distal part narrow with almost parallel sides and bearing an apical seta. Pleopod 2 (Fig. 4A) exopod triangular with convex outer margin and a small apical seta; endopod of two articles, longer than exopod, first article approx. three times longer than second, second article bearing distally rounded lobe and strong seta subapically cleft. Pleopod 3-5 exopods subtriangular with apical seta (Fig. 4B–D).

###### Distribution.

The species seems to be widely distributed in the central-eastern and south-eastern karstic areas of Sardinia. The species is mainly terrestrial even if it occasionally occurs also in water.

###### Remarks.

This species was described by Budde-Lund (1909) as Titanethes (Alpioniscus) fragilis from a Sardinian cave on material collected by Forsyth Major. No name for a specific cave was mentioned. However, considering that Forsyth Major discovered some troglobiotic species of different invertebrates during a digging campaign in caves of the Orosei Gulf, Casale et al. (2009) suggest that the cave explored by Forsyth Major most likely was the Grotta dell’Arciprete (= Grotta di Toddeitto) or one cave nearby in the same kastic area., e.g., Grotta del Bue Marino. The species is here redescribed on specimens from this last cave.

##### Alpioniscus thanit

Taxon classificationAnimaliaIsopodaTrichoniscidae

Taiti & Argano, 2009

Alpioniscus thanit Taiti & Argano, 2009: 39, figs 1–3; Taiti and Argano 2011: 169.

###### Type material re-examined.

**Prov. Nuoro**: 13 ♂♂, 29 ♀♀ paratypes (MZUF 9281), Dorgali, Cala Fuili, 40°15'27.8"N, 9°36'56.2"E, 25.IV.2008, leg. R. Argano and S. Taiti.

###### Material examined.

**Prov. Nuoro**: 3 ♂♂, 1 ♀ (MZUF 9812), Pozzo N.1 di Tres Puntas, c.n. 1150 Sa/NU, 40°22'44.76"N, 9°38'25.85"E, Monte Tuttavista, Galtellì, 2.VI.2013, leg. P. Marcia; 1 ♀ (MZUF 9813), same locality, 10.II.2013, leg. P. Marcia; 1 ♂, 4 ♀♀ (MZUF 9814), Grotta di Sos Jocos (or Grotta Taramelli), c.n. 344 Sa/NU, 40°19'04.30"N, 9°36'58.70"E, Su Anzu, Dorgali, 18.IX.2013, leg. P. Marcia and P. Nespoli; 1 ♀ (MZUF 9815), stessa località, 5.VI.2012, leg. P. Marcia; many ♂♂ and ♀♀ (MZUF 9816), same locality, 19.IV.2014, leg. P. Marcia, R. Argano and S. Taiti. **Prov. Ogliastra**: many ♂♂ and ♀♀ (MZUF 9808), Pedra Longa, Baunei, 40°01'35.4"N, 9°42'20.9"E, 20.V.2011, leg. R. Argano and S. Taiti; 1 ♂, 3 ♀♀ (MZUF 9809), same locality, 21.IV.2012, leg. R. Argano and S. Taiti; 1 ♂, 8 ♀♀ (MZUF 9810), Funtana Bausu, near Grotta S’erriu Mortu, Punta Giradili, Baunei, VI.2013, leg. C. Onnis; 1 ♂, 2 ♀♀ (MZUF 9811), Bacu Stirzili, Baunei , 23.II.2014, leg. C. Onnis.

###### Distribution.

The species is endemic to the area of Orosei Gulf, central-eastern Sardinia, where it occurs in both endogean and cave habitats.

##### Alpioniscus onnisi

Taxon classificationAnimaliaIsopodaTrichoniscidae

Taiti & Argano sp. n.

http://zoobank.org/A37ECC77-62C7-4650-B6D7-1D170683E4F8

**Figure 5.**
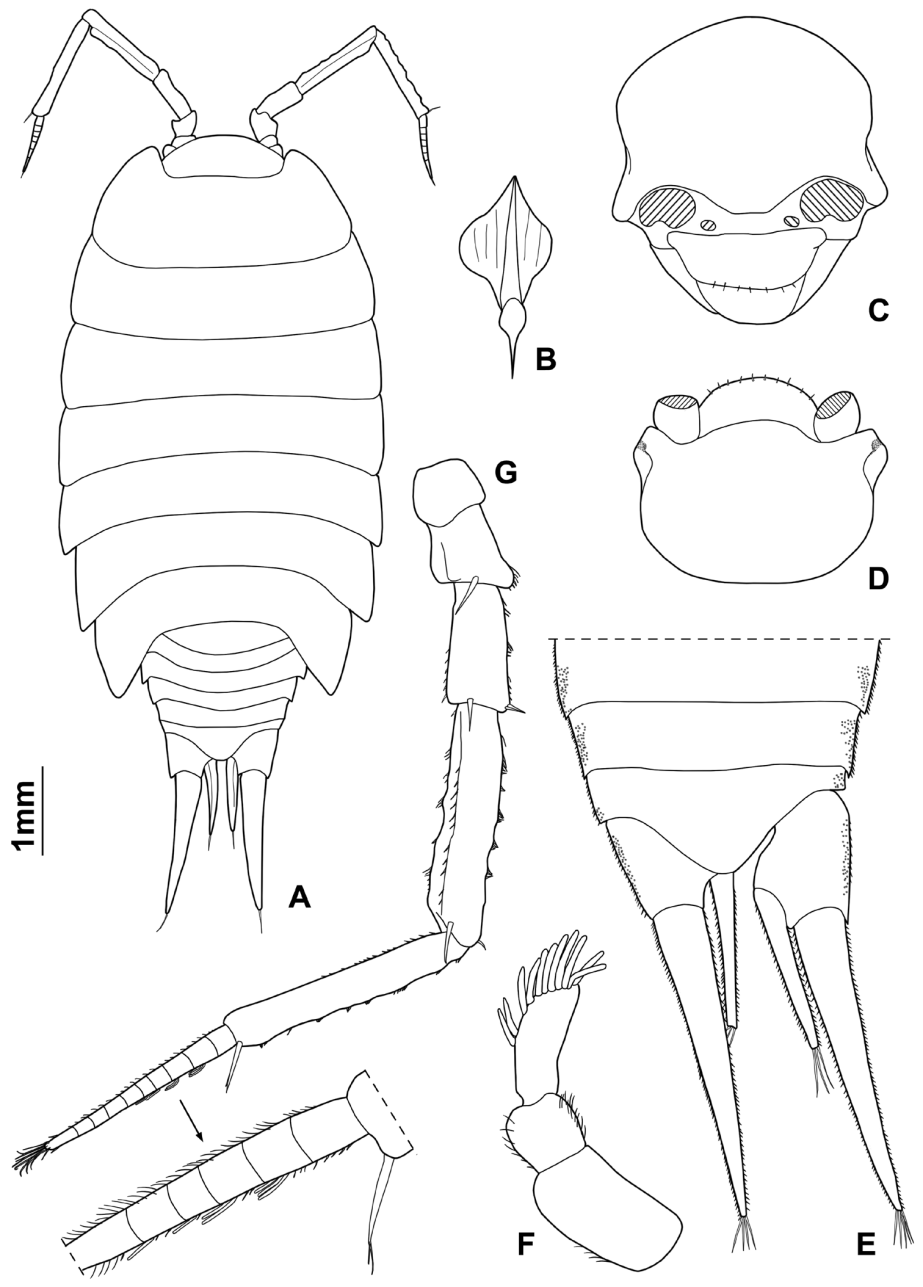
*Alpioniscusonnisi* Taiti & Argano, sp. n. from Grotta Giuanniccu Mene, ♂ paratype: **A** adult specimen, dorsal **B** dorsal scale-seta **C** cephalon, frontal **D** cephalon, dorsal **E** pleonites 4, 5, telson and uropods **F** antennula **G** antenna.

**Figure 6.**
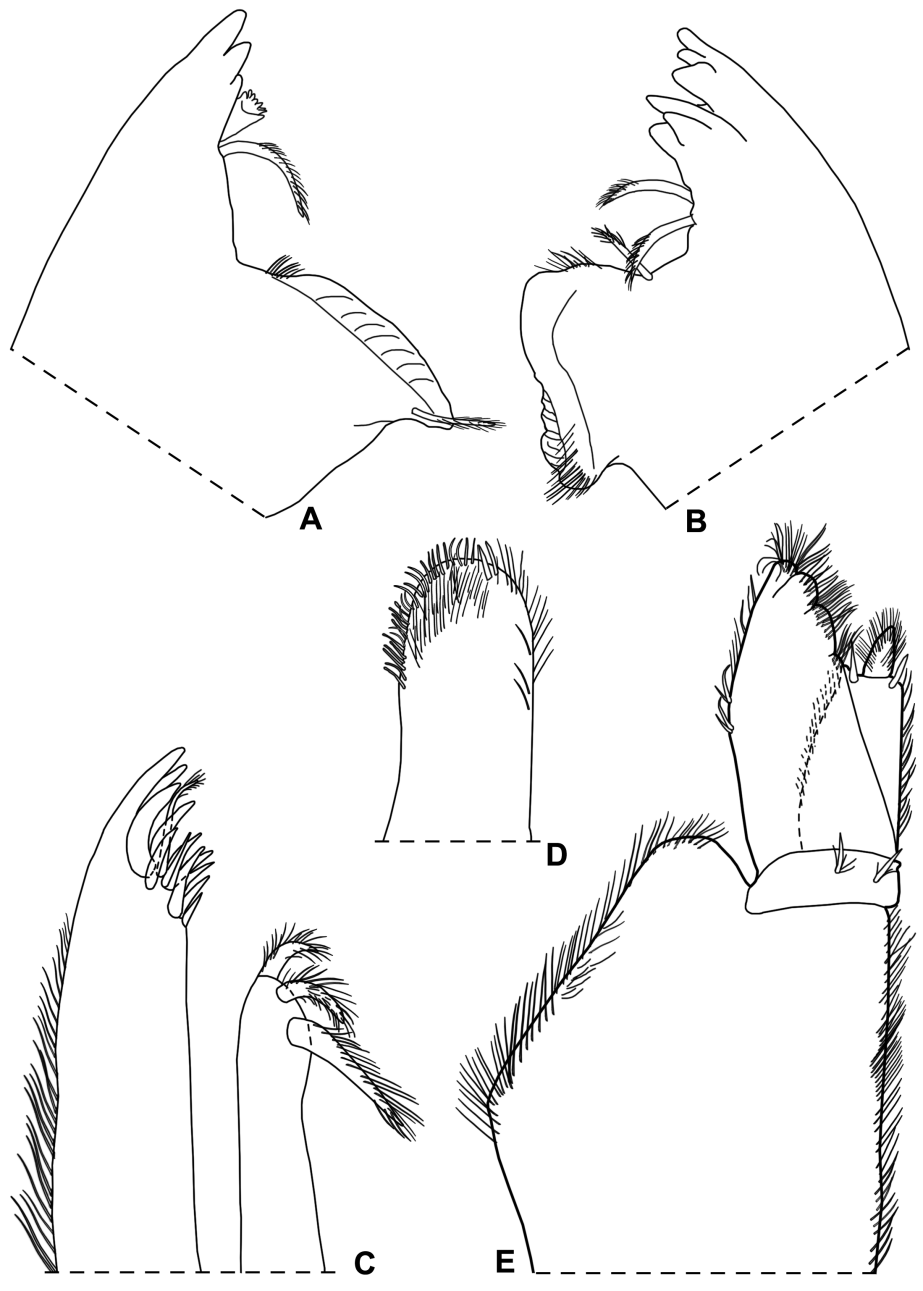
*Alpioniscusonnisi* Taiti & Argano, sp. n. from Grotta Giuanniccu Mene, ♂ paratype: **A** right mandible **B** left mandible **C** maxillula **D** maxilla **E** maxilliped.

**Figure 7.**
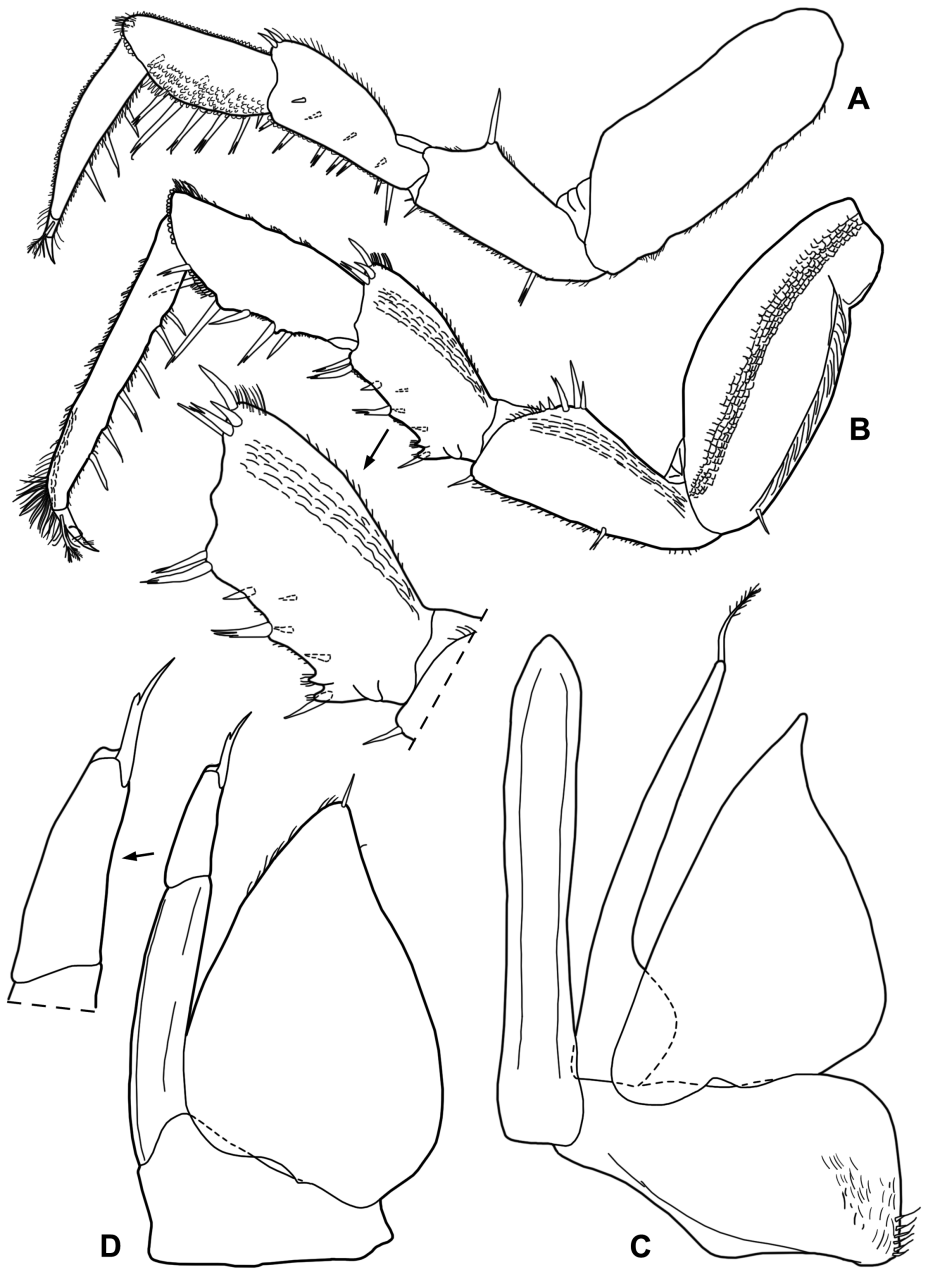
*Alpioniscusonnisi* Taiti & Argano, sp. n. from Grotta Giuanniccu Mene, ♂ paratype: **A** pereopod 1 **B** pereopod 7 **C** genital papilla and pleopod 1 **D** pleopod 2.

**Figure 8.**
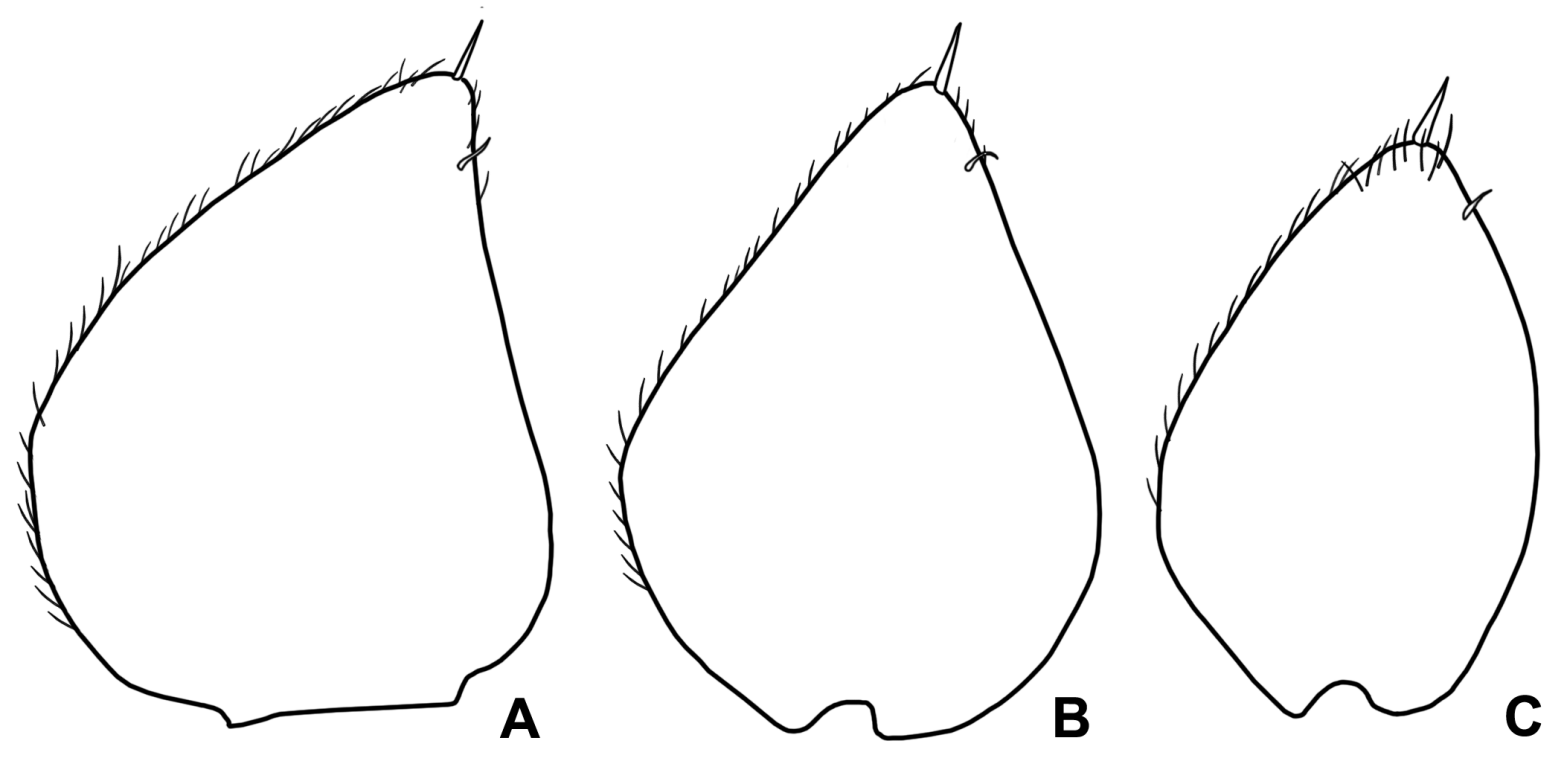
*Alpioniscusonnisi* Taiti & Argano, sp. n. from Grotta Giuanniccu Mene, ♂ paratype: **A** pleopod 3 exopod **B** pleopod 4 exopod **C** pleopod 5 exopod.

Figs 5 , 6 , 7 , 8 , 18 , 19

Alpioniscus fragilis ; Taiti and Argano 2011: 167 (partim: Grotta del Caprone Tyson).

###### Material examined.

**Prov. Cagliari**: *Holotype*: ♂ (MZUF 9817), Grotta Giuanniccu Mene, c.n. 735 Sa/CA, 39°31'32.3"N, 9°36'08.9"E, Monte Castello di Quirra, Villaputzu 20.IV.2012, leg. C. Onnis, S. Taiti, R. Argano. *Paratypes*: many ♂♂ and ♀♀ (MZUF 9817), same data as holotype; 1 ♀ (MZUF 9818), same locality, 8.I.2011, leg. C. Onnis; 4 ♂♂, 15 ♀♀, 2 juvs (MZUF 9819), same locality, 20.IV.2013, leg. C. Onnis, R. Argano and S. Taiti; 2 ♂♂, 4 juvs (MZUF 9820), same locality, 16.III.2012, leg. C. Onnis; 2 ♂♂, 7 ♀♀ (MZUF 9821), Grotta del Caprone Tyson, n.c.n., Monte del Castello di Quirra, Villaputzu, I.1999, leg. G. Marini; 1 ♂ juv. (MZUF 9822), “Prisoni” Ipogean Tomb, 39°31'24"N, 9°36'26"E, 15 m, Monte del Castello di Quirra, Villaputzu, 20.IV.2012, leg. C. Onnis, R. Argano and S. Taiti.

###### Description.

Maximum length: ♂, 7.5 mm; ♀, 9 mm. Colourless body, outline as in Fig. 5A. Dorsal surface smooth with lanceolate scale-setae as in Fig. 5B. Many gland pores on lateral margins of pleonites 4 and 5, telson and lateral surface of uropodal protopod (Fig. 5E). Cephalon (Fig. 5C, D) with suprantennal line medially blunt; antennal lobes quadrangular obliquely directed outwards with concave dorsal surface. Eyes absent. Posterior margin of pereonite 1-3 straight, and of pereonites 4–7 progressively more concave (Fig. 5A). Pleonites 3–5 with reduced posterior points (Fig. 5A, E). Telson (Fig. 5E) more than twice as wide as long; distal part triangular with concave sides and rounded apex. Antennula (Fig. 5F) with second article distinctly shorter than first and third; third article distally enlarged and bearing approx. ten apical aesthetascs. Antenna (Fig. 5G) with fifth article slightly longer than flagellum; flagellum of ten articles with four groups of aesthetascs. Mouth parts (Fig. 6A-E) as in *A.fragilis*. Pereopods with setose dactylar seta distally bifid, pereopod 7 with well developed water conducting system consisting of a groove with scales on basis, and lines of scales on ischium and merus. Uropod (Fig. 5E) with endopod distinctly shorter than exopod, endopod inserted proximally to exopod.

*Male*. Pereopod 1–4 (Fig. 7A) with carpus and merus bearing short scales on sternal margin. Pereopod 7 (Fig. 7B) ischium with straight sternal margin covered with short setae; merus with three lobes proximally. Genital papilla (Fig. 7C) with a rounded tip. Pleopod 1 (Fig. 7C) exopod triangular with acute apex; endopod enlarged at base, distal part narrow with almost parallel sides and bearing an apical seta. Pleopod 2 (Fig. 7D) exopod triangular with convex outer margin and a small apical seta; endopod with first article nearly twice as long as second, second article with a strong seta subapically cleft. Pleopod 3-5 exopods with a short apical seta (Fig. 8A–C).

###### Etymology.

The new species is named after our Sardinian friend Carlo Onnis for his enthusiastic and efficient activity in collecting subterranean fauna, including part of the material treated here.

###### Remarks.

*Alpioniscusonnisi* sp. n. is very similar to *A.fragilis* and *A.thanit*. It differs from the former in the smooth instead of granulated dorsal surface, the shape of the dorsal scale-setae, telson with triangular instead of trapezoidal distal part, and the male pleopod 2 endopod lacking a subapical lobe; from the latter mainly in the larger body shape and the male pleopod 1 exopod with slightly convex, instead of concave, medial margin, and shorter distal part.

##### Alpioniscus kuehni

Taxon classificationAnimaliaIsopodaTrichoniscidae

(Schmalfuss, 2005) comb. n.

**Figure 9.**
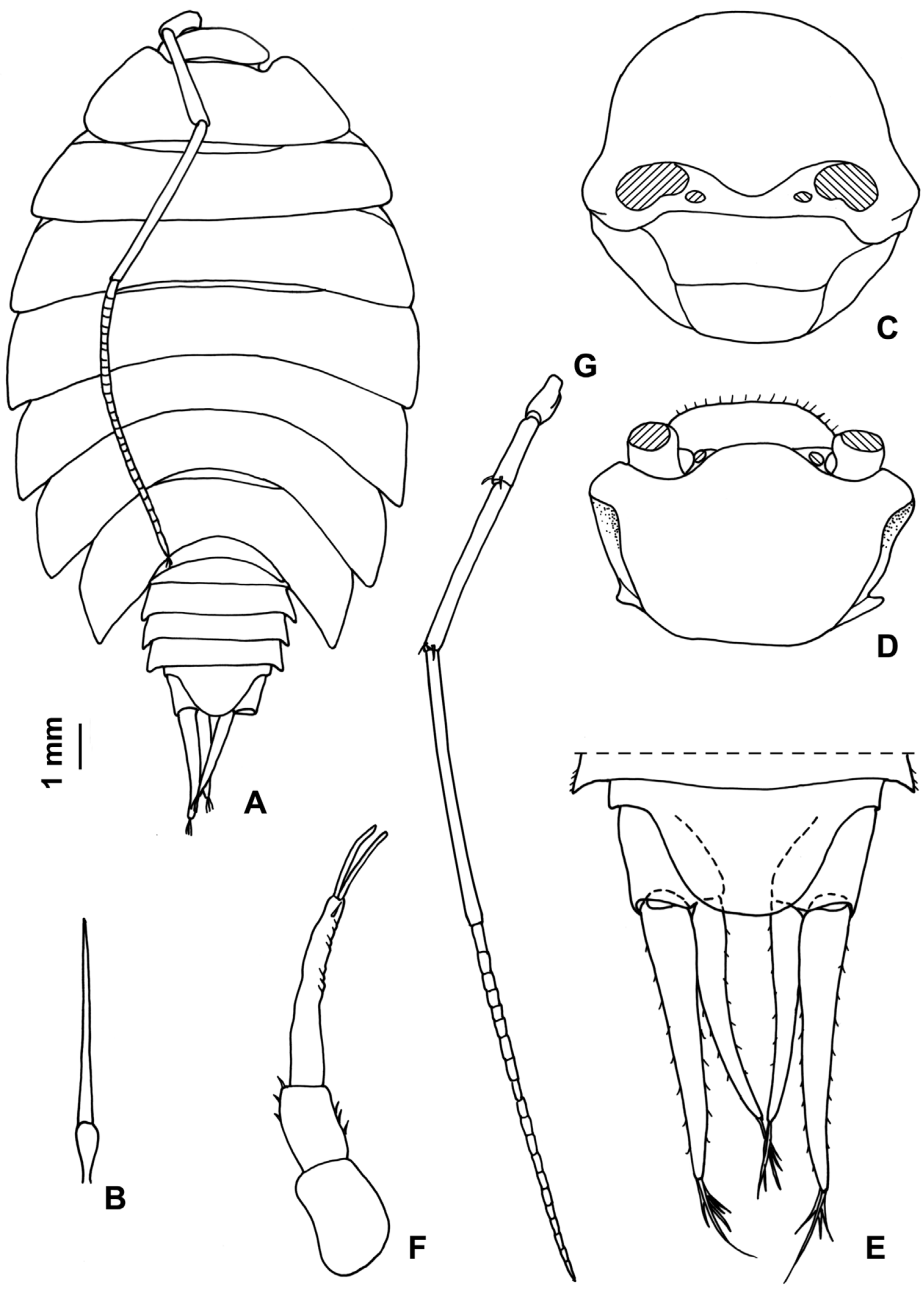
*Alpioniscuskuehni* (Schmalfuss, 2005), comb. n. from Grotta del Bue Marino, ♀: **A** adult specimen, dorsal **B** dorsal scale-seta **C** cephalon, frontal **D** cephalon, dorsal **E** telson and uropods **F** antennula **G** antenna.

**Figure 10.**
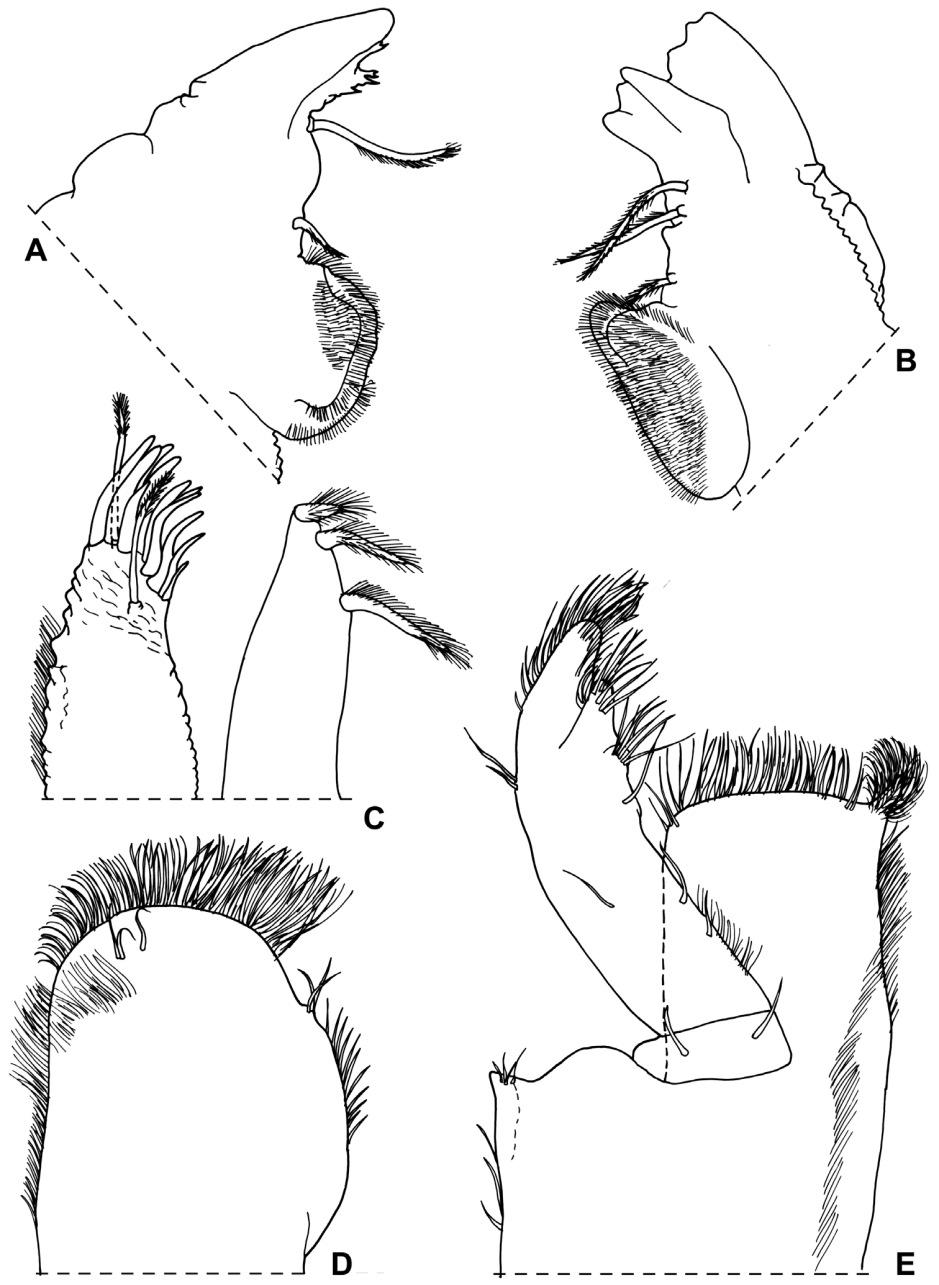
*Alpioniscuskuehni* (Schmalfuss, 2005), comb. n. from Grotta del Bue Marino, ♀: **A** right mandible **B** left mandible **C** maxillula **D** maxilla **E** maxilliped.

**Figure 11.**
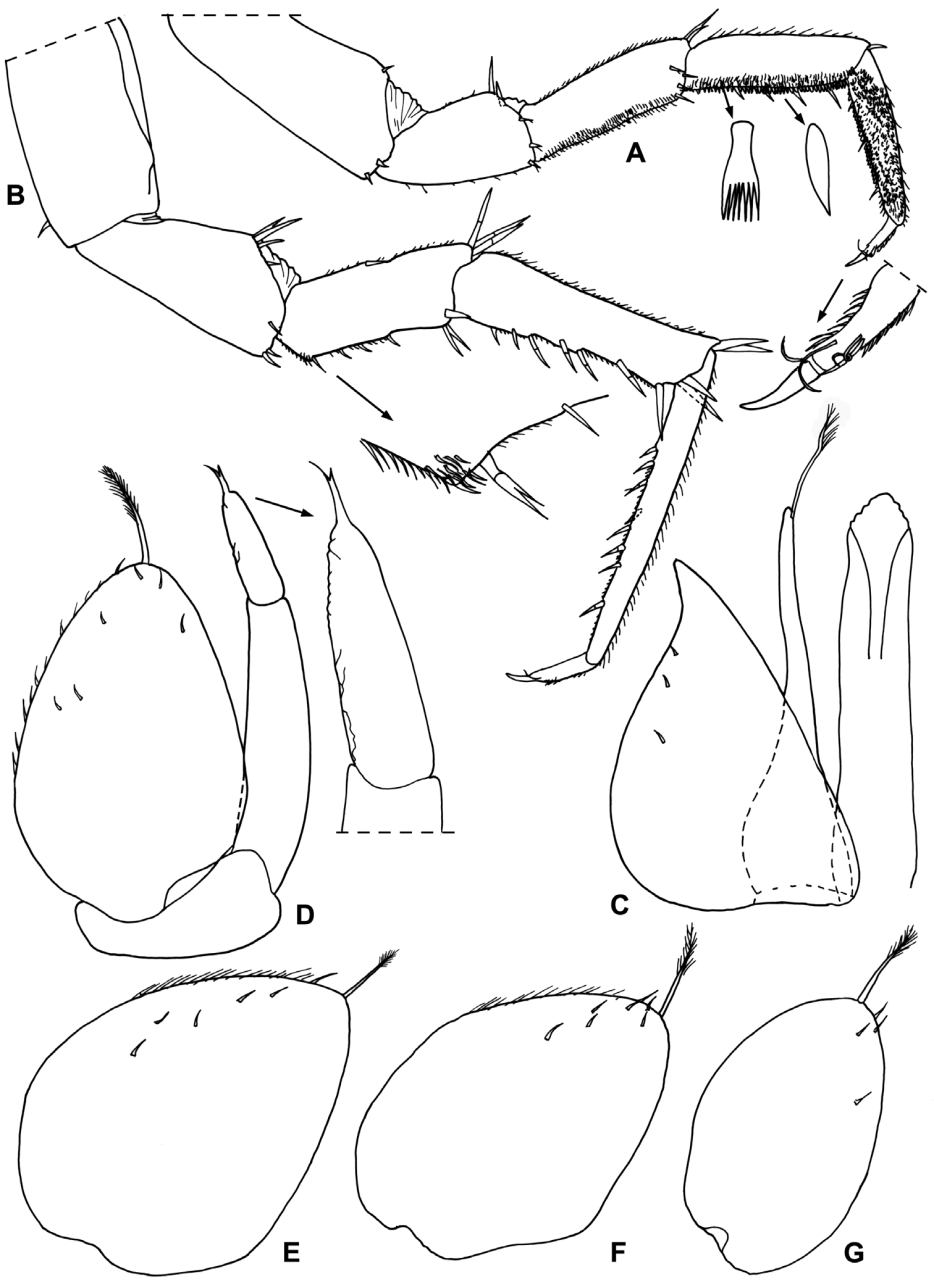
*Alpioniscuskuehni* (Schmalfuss, 2005), comb. n. from Grotta del Bue Marino, ♂: **A** pereopod 1 **B** pereopod 7 **C** genital papilla and pleopod 1 **D** pleopod 2 **E** pleopod 3 exopod **F** pleopod 4 exopod **G** pleopod 5 exopod.

Figs 9 , 10 , 11 , 18 , 19

Utopioniscus kuehni ; Schmalfuss 2005: 2, figs 1–5, 7–35; Oertel and Patzner 2007: 62, 64, fig. 4; Taiti and Argano 2011: 166.

###### Material examined.

**Prov. Nuoro**: 4 ♂♂, 3 ♀♀ (MZUF 9827), Grotta del Bue Marino, c.n. 12 Sa/NU, 40°14'55.72"N, 9°37'24.80"E, Cala Gonone, Dorgali, 25.IV.2012, leg. E. Dallocchio and P. Marcia; 1 ♂, 1 juv. (MZUF 9828), same locality, 10.IX.2006, P. Marcia and F. Stoch; 11 ♂♂, 4 ♀♀ (MZUF 9829), Grotta Su Bentu, c.n. 105 Sa/NU, 40°15'18.23"N, 9°29'6.52"E, Lanaittu, Oliena, 6.I.2013, leg. P. Marcia; 2 ♂♂ (MZUF 9830), same locality, 11-14.IX.2012, leg. P. Marcia and Astronauts; 1 ♀ (MZUF 9831), same locality, 21.IX.2013, leg. Astronauts; 1 ♂, 2 ♀♀ (MZUF 9832), same locality, 15.IX.2014, leg. Astronauts.

###### Distribution.

The species is endemic to karstic areas of Supramonte, central-eastern Sardinia.

###### Remarks.

*Alpioniscuskuehni* was originally described as *Utopioniscuskuehni* gen. n., sp. n. by Schmalfuss (2005) on specimens collected in underground waters from two caves on the central-eastern coast of Sardinia, Grotta dell’Utopia and Grotta del Bel Torrente, with entrances at 30 m and 16 m below sea level, respectively. These caves are the estuaries of subterranean streams which open up in the sea (De Waele and Forti 2003). In the Grotta del Bel Torrente the specimens were collected 700 m inland, where water salinity was 1% (Schmalfuss 2005; Oertel and Patzner 2007). In the Grotta dell’Utopia they were collected 2000 m inland together with a species of the freshwater stygobiotic *Stenasellus* Dollfus, 1897 (Asellota, Stenasellidae). The specimens from Grotta del Bue Marino and Grotta Su Bentu here examined were collected in fresh water lakes and are morphologically identical to the specimens from the type localities. This fact confirms that *A.kuehni* is a freshwater stygobiotic species.

The species is well described by Schmalfuss (2005) and is here fully illustrated on specimens from Grotta del Bue Marino (Figs 9–11). As confirmed by molecular data (see below), the genus *Utopioniscus* must be considered to be a junior synonym of *Alpioniscus*. In fact, all the diagnostic morphological characters of the genus *Alpioniscus* mentioned by Vandel (1960) are present also in *A.kuehni*, in particular the shape of the male pleopods 1 and 2, as already pointed out by Schmalfuss (2005). *Alpioniscuskuehni* is mainly characterized by the enlarged shape of the pereon, the reduction of the number of aesthetascs of the antennula, the very thin antenna with the flagellum of 20 to 30 articles, the enlargement of the maxillipedal endite, and the lack of groove with scales on the pereopod 7 for the water conducting system. The last two characters are certainly adaptive for aquatic life. The enlarged maxillipedal endite is, in fact, present also in the following two new species which are both aquatic.

##### Alpioniscus stochi

Taxon classificationAnimaliaIsopodaTrichoniscidae

Taiti & Argano sp. n.

http://zoobank.org/E76BDBF8-0FE5-4DEF-BAF1-7CA0C3F92F34

**Figure 12.**
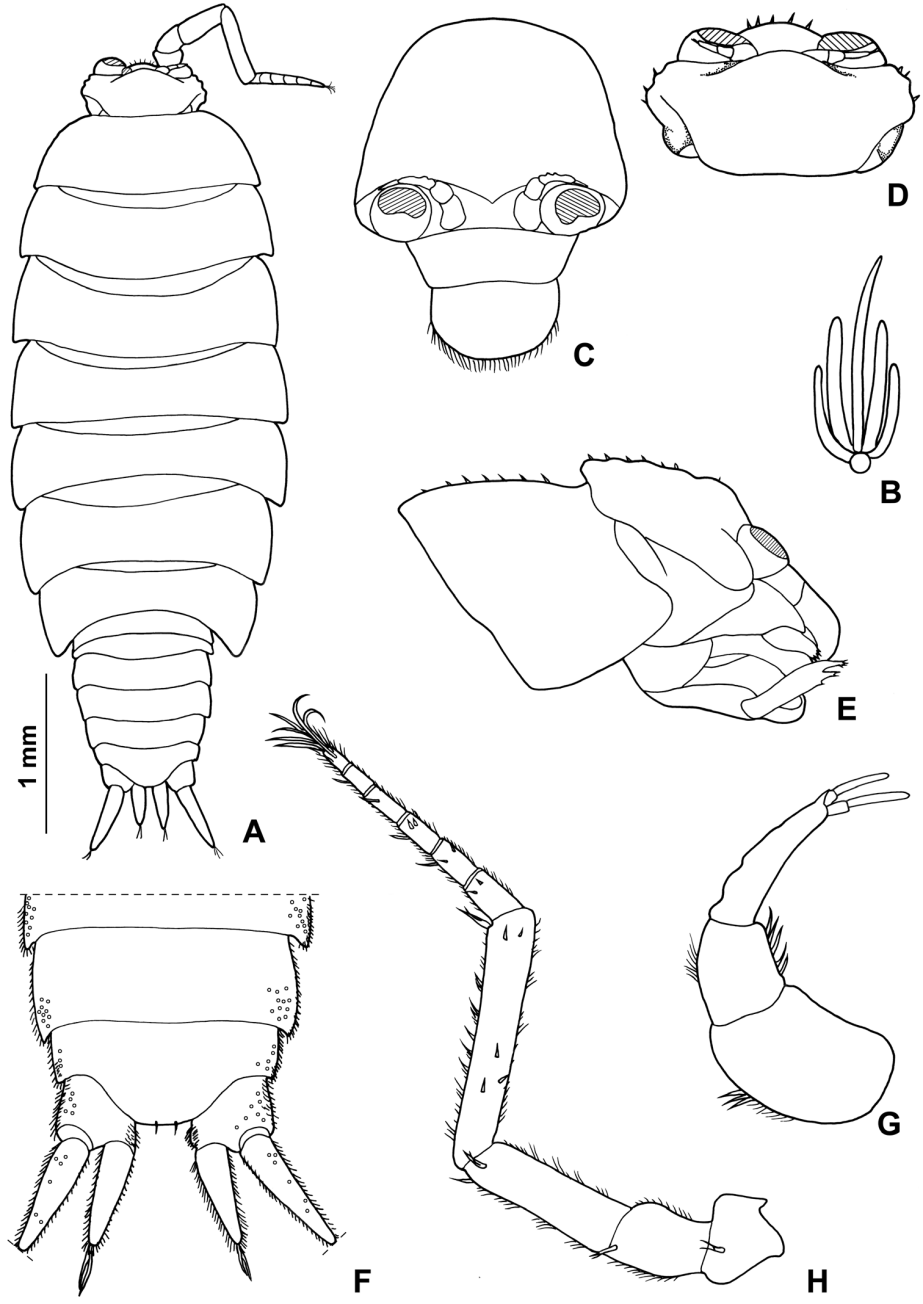
*Alpioniscusstochi* Taiti & Argano, sp. n. from Grotta Su Palu, ♂ paratype: **A** adult specimen, dorsal **B** dorsal scale-seta **C** cephalon, frontal **D** cephalon, dorsal **E** cephalon and pereonite 1, lateral **F** pleonites 4, 5, telson and uropods **G** antennula **H** antenna.

**Figure 13.**
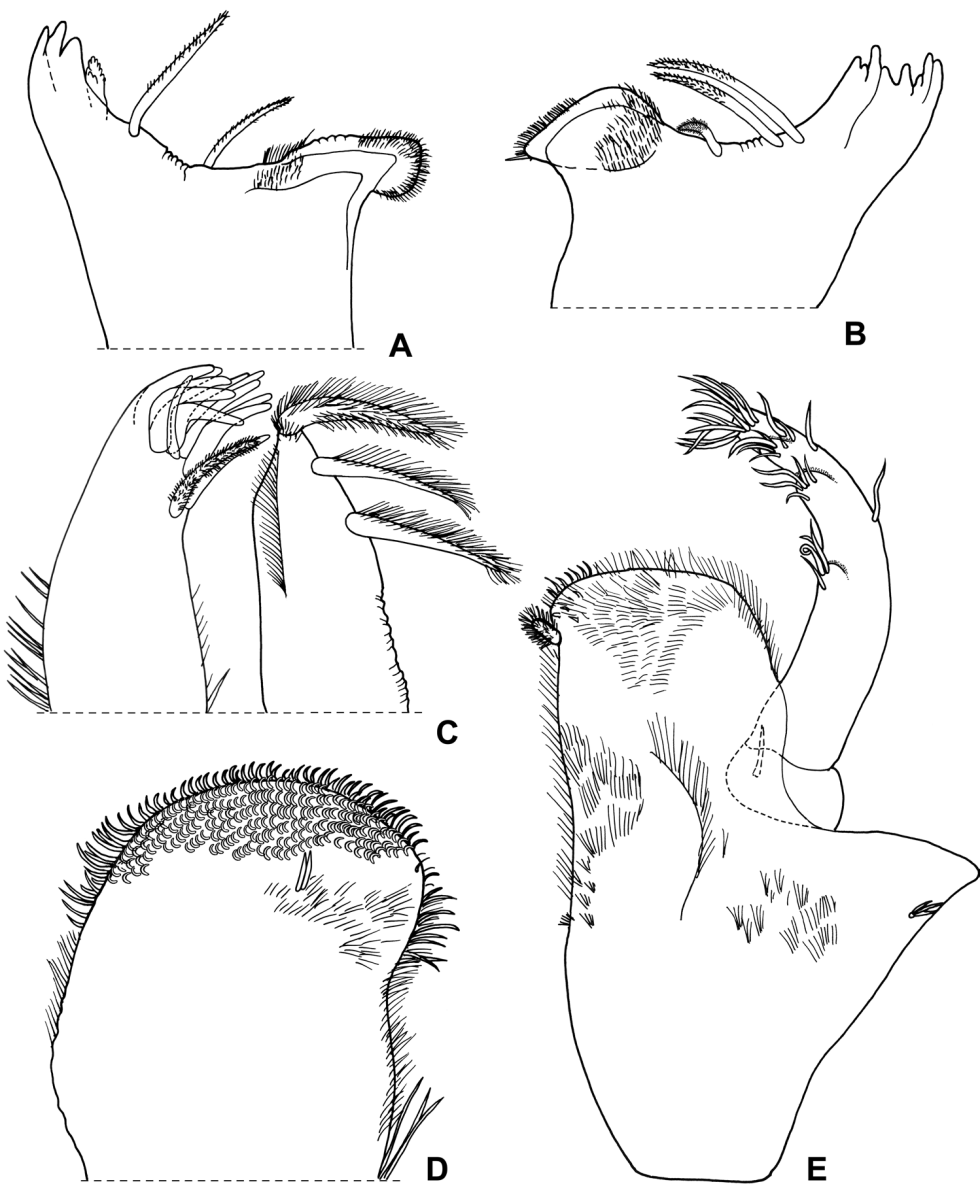
*Alpioniscusstochi* Taiti & Argano, sp. n. from Grotta Su Palu, ♂ paratype: **A** right mandible **B** left mandible **C** maxillula **D** maxilla **E** maxilliped.

**Figure 14.**
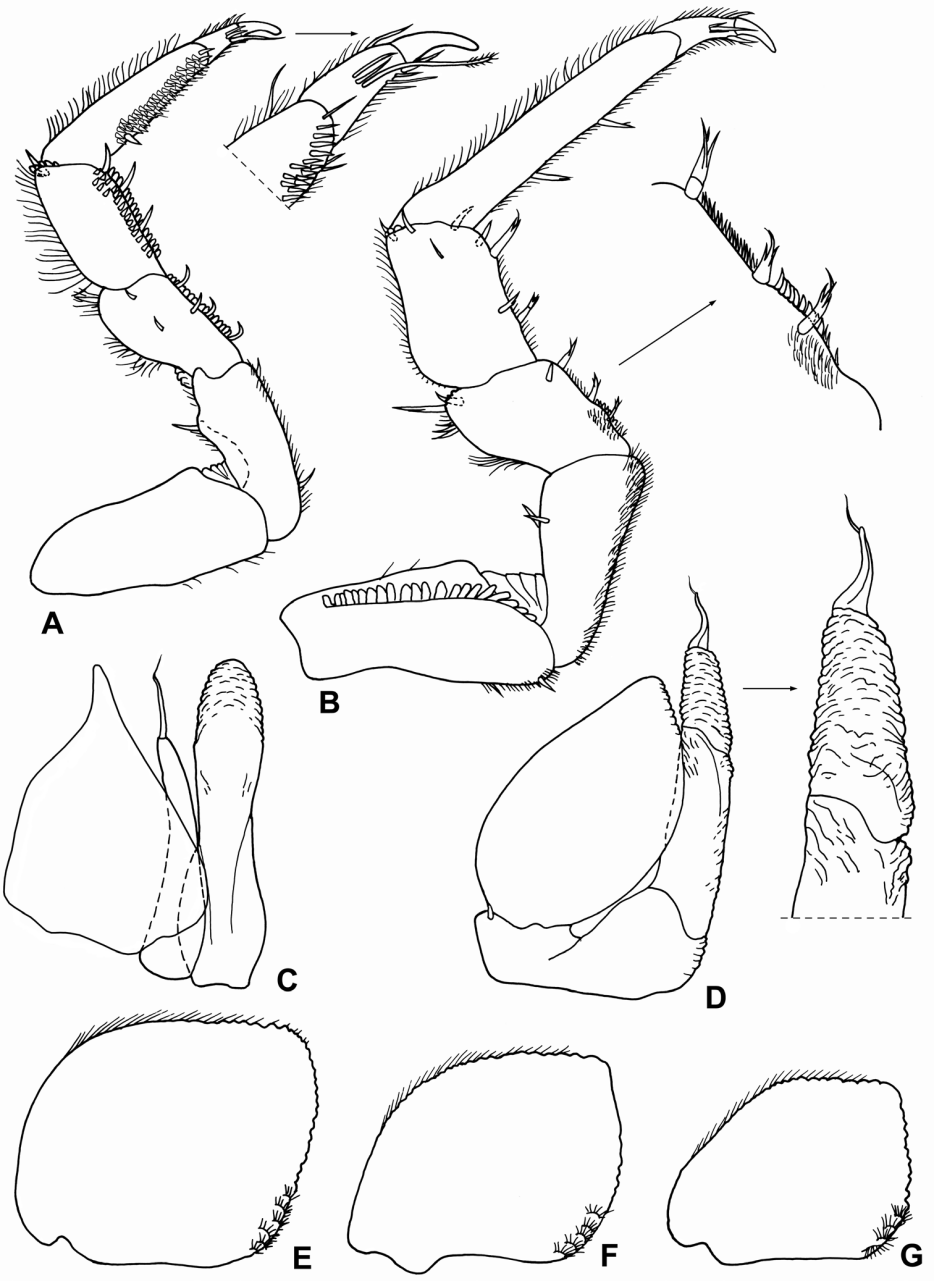
*Alpioniscusstochi* Taiti & Argano, sp. n. from Grotta Su Palu, ♂ paratype: **A** pereopod 1 **B** pereopod 7 **C** genital papilla and pleopod 1 **D** pleopod 2 **E** pleopod 3 exopod **F** pleopod 4 exopod **G** pleopod 5 exopod.

Figs 12 , 13 , 14 , 18 , 19

###### Material examined.

**Prov. Ogliastra**: *Holotype*: ♂ (MZUF 9833), Grotta Su Palu, c.n. 1988 Sa/OG, 40°10'38.23"N, 9°33'50.53"E, 185 m, Codula Ilune, Urzulei, 1.V.2009, leg. F. Stoch and G. Tomasin. *Paratypes*: 3 ♂♂, 4 ♀♀ (MZUF 9833), same data as holotype; 4 ♂♂, 5 ♀♀ (MZUF 9834), same locality, 18.IX.2010, leg. G. Tomasin.

###### Description.

Maximum length: ♂ and ♀, 4.5 mm. Colourless body, pleon slightly narrower than pereon (Fig. 12A). Dorsal surface smooth with some scattered scale-setae as in Fig. 12B. Some gland pores on lateral margins of pleonites 4 and 5, telson and on dorsal surface of uropodal protopod and exopod (Fig. 12F). Cephalon (Fig. 12C–E) with suprantennal line V-shaped; antennal lobes quadrangular. Eyes absent. Posterior margins of pereonite 1-4 straight, of pereonites 5–7 progressively more concave (Fig. 12A). Pleonites 3–5 with very short posterior points (Fig. 12F). Distal part of telson with concave sides and very broadly rounded apex (Fig. 12F). Antennula (Fig. 12G) with distal article narrow and bearing two apical aesthetascs. Antenna (Fig. 12H) with fifth article as long as flagellum; flagellum of five to seven articles. Mandibles with two penicils in the right (Fig. 13A) and three penicils in the left (Fig. 13B). Outer branch of maxillula with 5 + 6 teeth, apically entire, three or four of the outer group strongly bent inwards, two slender stalks, one setose; inner branch with three long penicils (Fig. 13C). Maxilla with setose apex (Fig. 13D). Maxilliped (Fig. 13E) endite quadrangular with a setose distal margin and a distinct subapical short penicil on medial margin; palp narrow and bent in medial direction, three tufts of setae on medial margin and one apically, basal article with a single seta; basis distally enlarged with a triangular lobe on outer margin. Pereopods with setose dactylar seta (Fig. 14A). Pereopod 7 (Fig. 14B) basis with a groove covered with scales on rostral surface (water conducting system). Uropod (Fig. 12F) with protopod not grooved on outer margin; endopod slightly shorter than exopod, exopod and endopod inserted at the same level.

*Male*. Pereopod 1–4 (Fig. 14A) with carpus and merus bearing numerous short scales on sternal margin. Pereopod 7 (Fig. 14B) ischium with straight sternal margin covered with short setae; merus with scales on sternal margin, carpus enlarged proximally. Genital papilla (Fig. 14C) with a rounded tip. Pleopod 1 (Fig. 14C) exopod triangular with narrow posterior point; endopod narrow with almost parallel sides, armed with an apical seta. Pleopod 2 (Fig. 14D) exopod subovoidal, with no apical seta; endopod with first article nearly twice as long as second, strong terminal seta ending with thinner seta. Pleopods 3-5 exopods (Fig. 14E–G) quadrangular with no apical seta.

###### Etymology.

The new species is named after our colleague and friend Dr. Fabio Stoch, who greatly contributed to the knowledge of Italian stygobiotic fauna and collected part of the material.

###### Remarks.

The specimens were collected under stones on the bottom of a subterranean stream in the Grotta Su Palu, in the eastern part of the karstic area of Supramonte. *Alpioniscusstochi* sp. n. is similar to *A.kuehni* in having a thin third article of the antennula bearing a small number of aesthetascs, and the maxilliped with a quadrangular endite and a narrow palp bent medially. It is readily distinguishable from *A.kuehni* by the smaller size (4.5 mm vs. 12.0 mm of the latter), narrower body shape, shorter and more thickset antenna with smaller number of flagellar articles, uropods with shorter branches, the presence of a water conducting system on pereopod 7 ischium, a more thickset merus of the male pereopod 7, a stouter male pleopod 2 endopod, and no apical seta on the exopod of the male pleopods 2-5.

##### Alpioniscus sideralis

Taxon classificationAnimaliaIsopodaTrichoniscidae

Taiti & Argano sp. n.

http://zoobank.org/06FC60AA-B680-4684-B5A3-1FF995C44254

**Figure 15.**
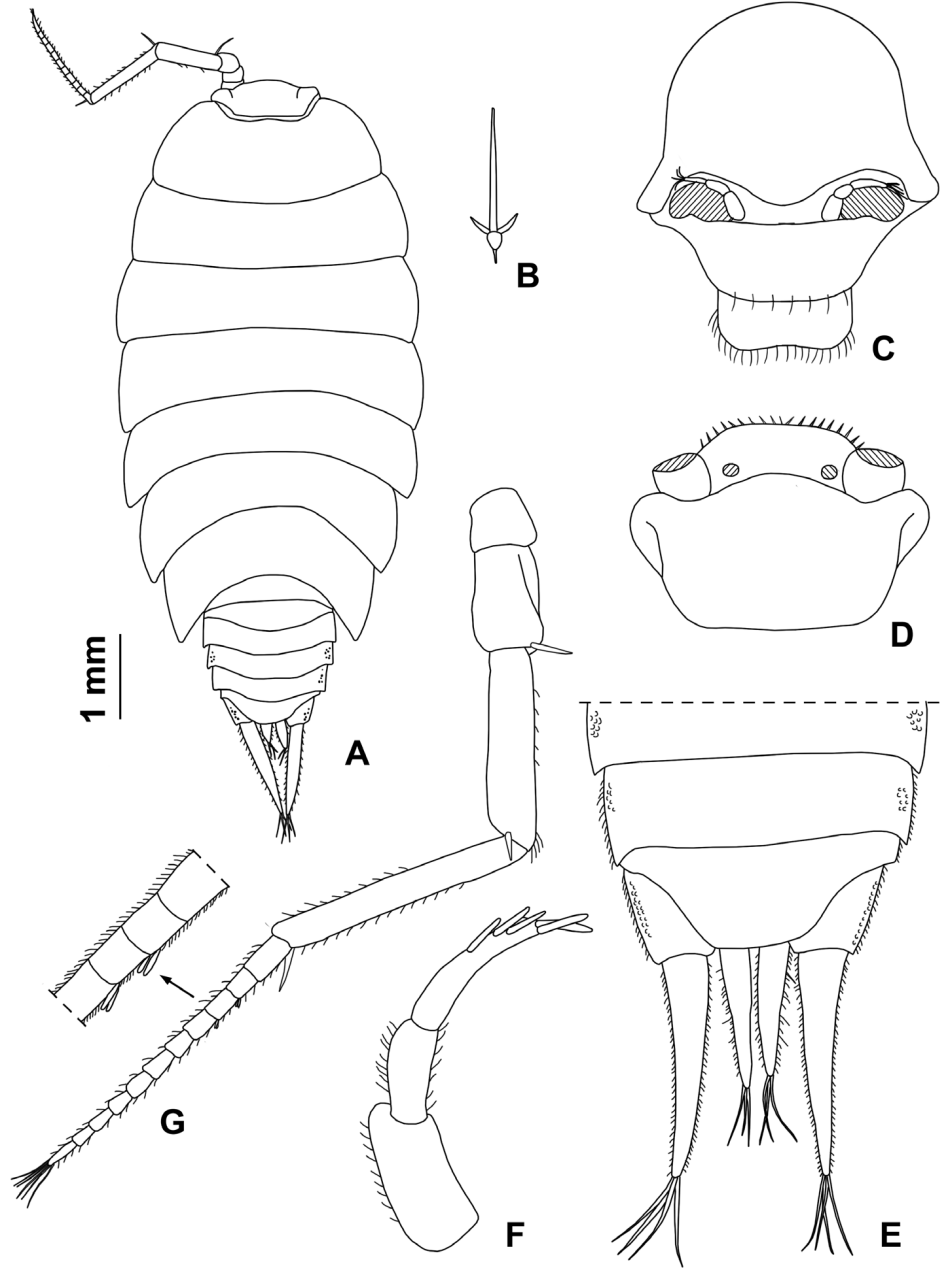
*Alpioniscussideralis* Taiti & Argano, sp. n. from Grotta Su Bentu, ♂ paratype: **A** adult specimen, dorsal **B** dorsal scale-seta **C** cephalon, frontal **D** cephalon, dorsal **E** pleonites 4, 5, telson and uropods **F** antennula **G** antenna.

**Figure 16.**
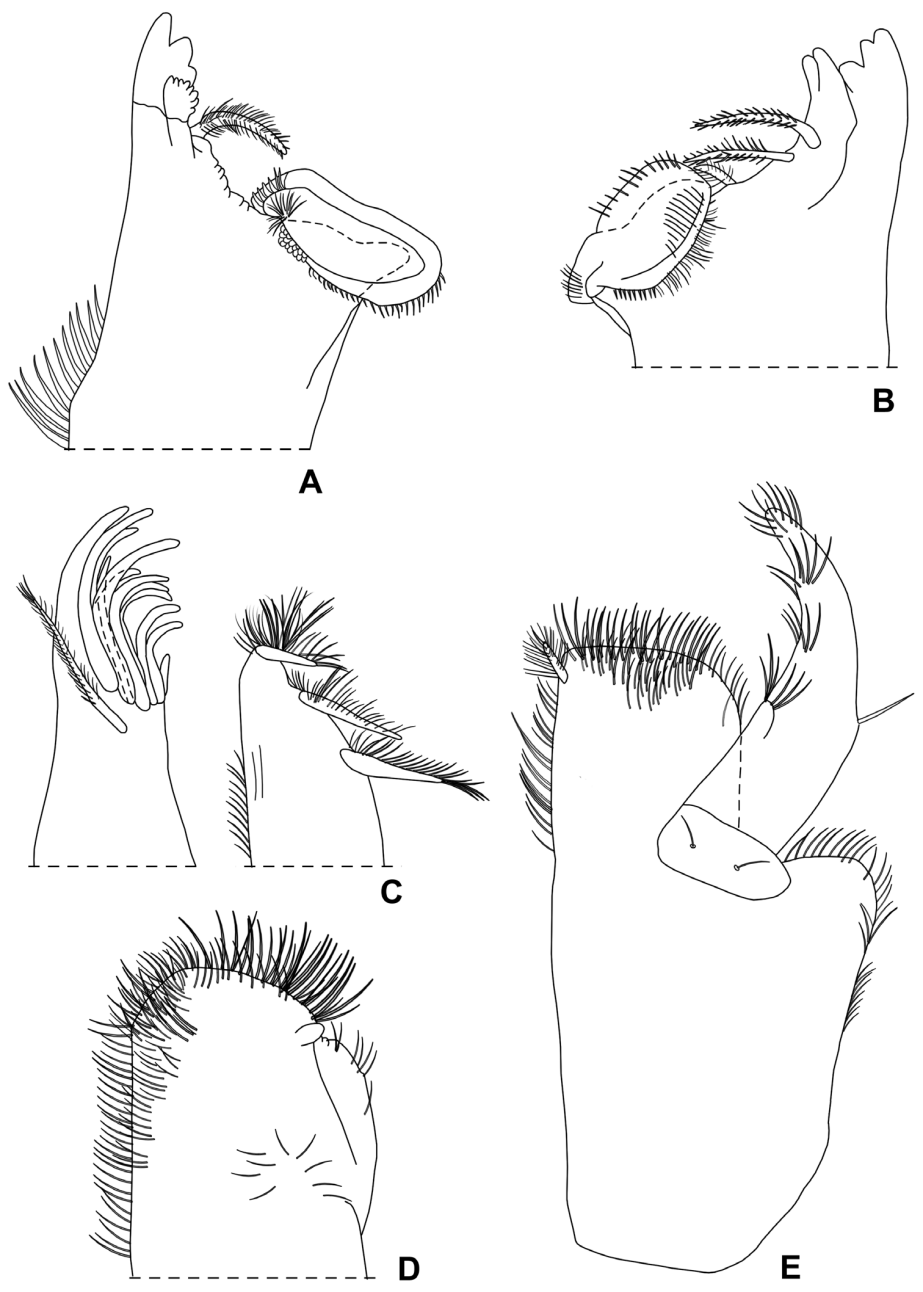
*Alpioniscussideralis* Taiti & Argano, sp. n. from Grotta Su Bentu, ♂ paratype: **A** right mandible **B** left mandible **C** maxillula **D** maxilla **E** maxilliped.

**Figure 17.**
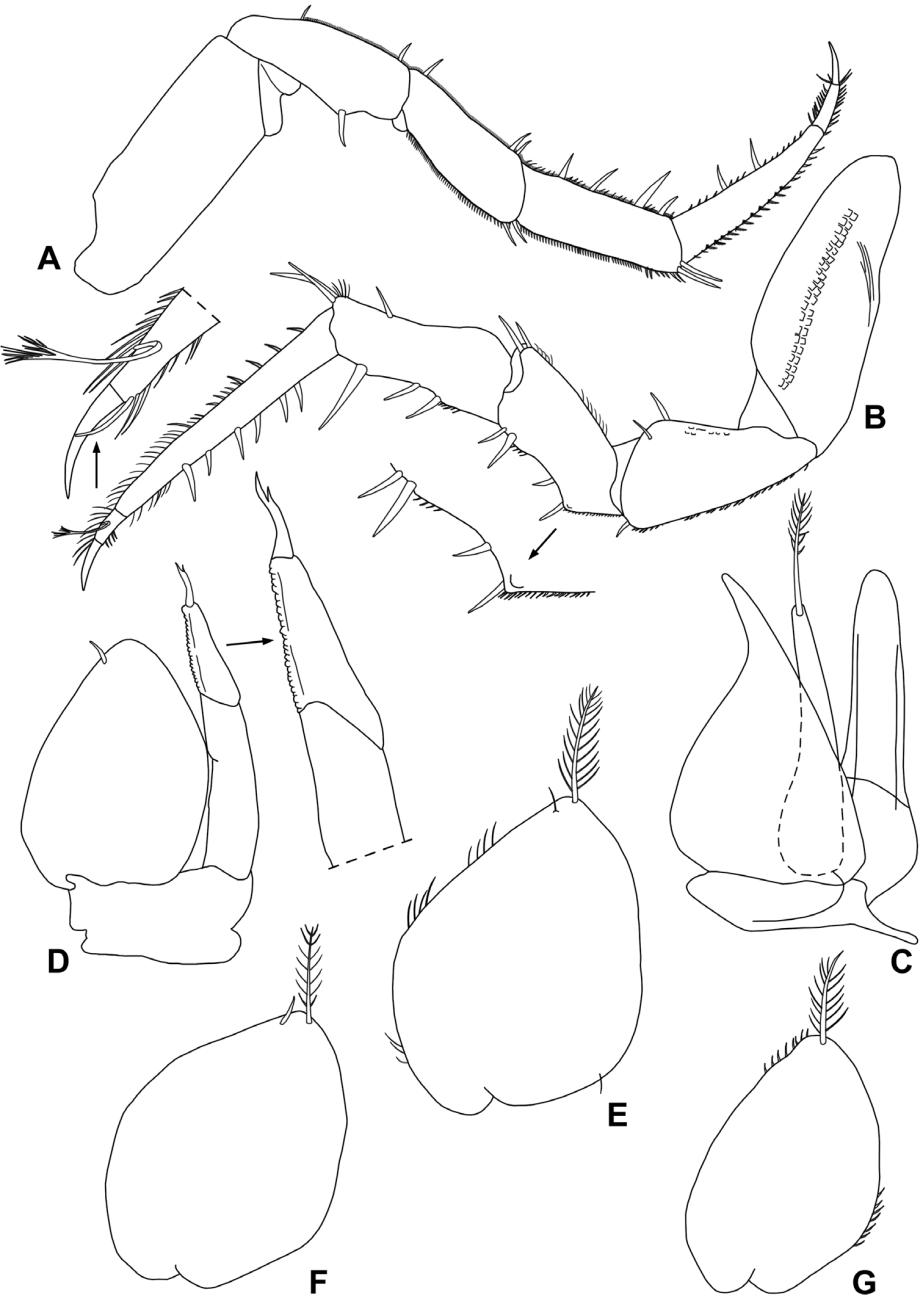
*Alpioniscussideralis* Taiti & Argano, sp. n. from Grotta Su Bentu, ♂ paratype: **A** pereopod 1 **B** pereopod 7 **C** genital papilla and pleopod 1 **D** pleopod 2 **E** pleopod 3 exopod **F** pleopod 4 exopod **G** pleopod 5 exopod.

**Figure 18.**
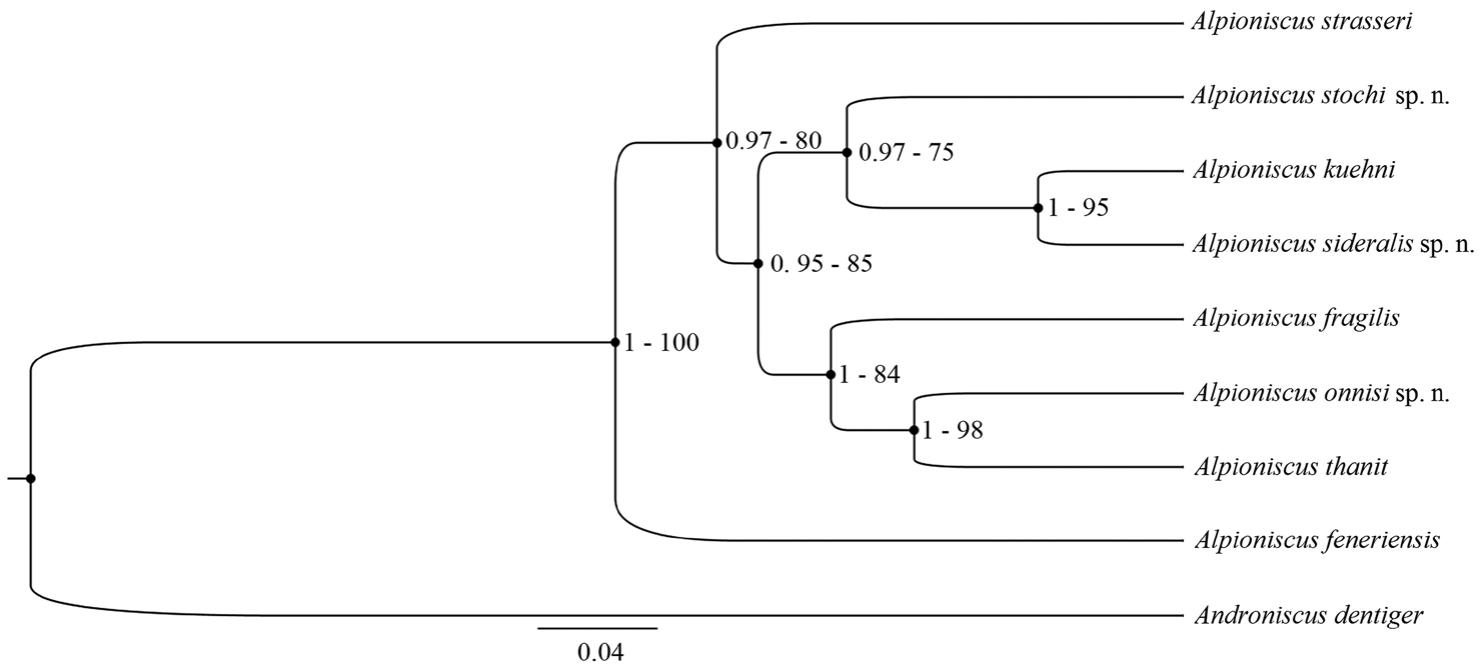
Gene tree according to BI showing the interrelationships among species based on COI. The branch length scale refers to the number of substitutions per site. Nodal supports are BI posterior probabilities (the first value), and ML bootstrap values (the second value).

**Figure 19.**
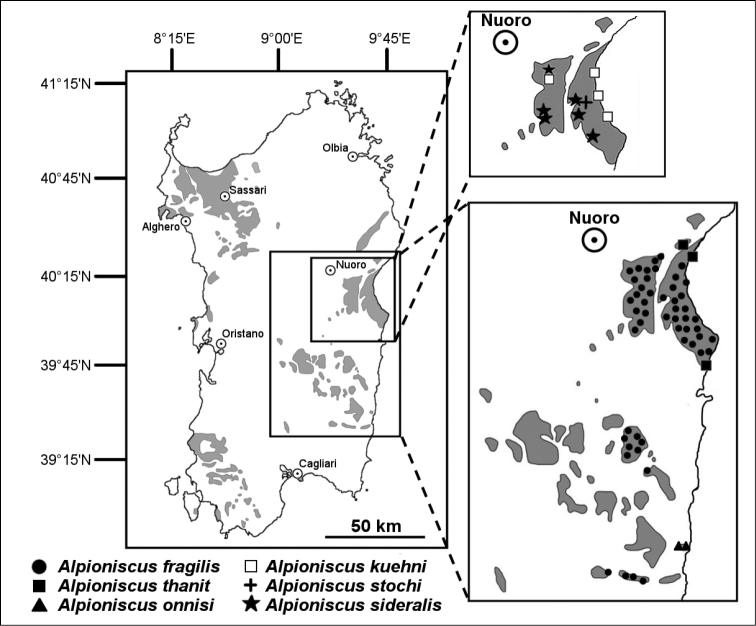
Distribution map of *Alpioniscus* species in Sardinia (after Taiti and Argano 2011 and new data).

Figs 15 , 16 , 17 , 18 , 19

Alpioniscus n. sp.; Bessone et al. 2013: 325, fig. 11; 2016: 218, fig. 8.

###### Material examined.

**Prov. Nuoro**: *Holotype*: ♂ (MZUF 9835), Grotta Su Bentu, c.n. 105 Sa/NU, 40°15'18.23"N, 9°29'6.52"E, Lanaittu, Oliena, 21.IX.2013, leg. Astronauts. *Paratypes*: 1 ♂, 1 ♀ (MZUF 9836), same data as holotype; 1 ♂, 3 ♀♀ (MZUF 9836), same locality, 11-14.IX.2012, leg. P. Marcia and Astronauts; 1 ♂, 2 ♀♀ (MZUF 9837), same locality, 6.I.2013, leg. P. Marcia; 2 ♂♂, 1 ♀ (MZUF 9838), Grotta Sas Venas, c.n. 3064 Sa/NU, 40°07'32.33"N, 9°26'27.04"E, Orgosolo, VI.2013, leg. P. Marcia; 3 ♀♀ (MZUF 9839), Grotta Piggio de Janas, c.n. 3236 Sa/NU, 40°08'32.70"N, 9°27'18.60"E, Tauledda, Codula del Flumineddu, Orgosolo, 28.X.2012, leg. C. Corongiu. **Prov. Ogliastra**: 2 ♂♂, 1 ♀ (MZUF 9840), Grotta Lovettecannas, c.n. 2642 Sa/OG, 40°08'33.72"N, 9°34'35.35"E, Baunei, 5.I.2013, leg. L. Sanna; 7 ♂♂, 7 ♀♀ (MZUF 9841), same locality, 1.IV.2013, leg. P. Marcia; 1 ♂ (MZUF 9842), Grotta Su Palu, c.n. 1988 Sa/OG, 40°10'38.23"N, 9°33'50.53"E, 185 m, Codula Ilune, Urzulei, 8.XII.2012, leg. P. Marcia; 5 ♂♂, 9 ♀♀ (MZUF 9843), Grotta Istirzili, c.n. 50 Sa/OG, 40°04'49.50"N, 9°37'13.40"E, Baunei, 12.V.2013, leg. C. Onnis.

###### Description.

Maximum length: ♂, 7 mm; ♀, 7.5 mm. Body colourless, ovoidal, with pleon narrower than pereon (Fig. 15A). Dorsal surface smooth with some scattered scale-setae as in Fig. 15B. Some gland pores on lateral margins of pleonites 4 and 5 and on uropodal protopods (Fig. 15E). Cephalon (Fig. 15C, D) with suprantennal line sinuous; antennal lobes quadrangular. Eyes absent. Posterior margin of pereonite 1-3 straight, of pereonites 4–7 progressively more concave (Fig. 15A). Pleonites 3–5 with very short posterior points (Fig. 15E). Distal part of telson with slightly concave sides and truncate apex (Fig. 15E). Antennula (Fig. 15F) with distal article narrow and bearing two apical and three subapical aesthetascs. Antenna (Fig. 15G) with fifth article of peduncle shorter than flagellum; flagellum of 10-11 articles with couple of aesthetascs on second and third article. Mandibles with one penicil in the right (Fig. 16A) and three penicils in the left (Fig. 16B). Outer branch of maxillula with 5 + 6 teeth, apically entire, and two slender setose stalks; inner branch with three penicils increasing in length from distal to proximal (Fig. 16C). Maxilla with setose and bilobed apex, inner lobe very small (Fig. 16D). Maxilliped (Fig. 16E) endite quadrangular with setose distal margin and distinct subapical penicil on medial margin; palp narrow and bent in medial direction, with three tufts of setae on medial margin, tuft at apex and single seta on outer margin, basal article with two setae; basis distally enlarged with a rounded lobe on outer margin. Pereopods with bifid setose dactylar seta (Fig. 17A). Pereopod 7 (Fig. 17B) basis with water conducting system. Uropod (Fig. 15E) with protopod not grooved on outer margin; endopod distinctly shorter than exopod, exopod inserted slightly distally to endopod.

*Male*. Pereopod 1–4 (Fig. 17A) with ischium, carpus and merus bearing numerous short scales on sternal margin. Pereopod 7 (Fig. 17B) ischium with straight sternal margin covered with short setae; merus with triangular lobe at base of sternal margin, more or less protruding according to size; carpus enlarged proximally. Genital papilla (Fig. 17C) with a rounded tip. Pleopod 1 (Fig. 17C) exopod triangular with long narrow distal point slightly bent outwards; endopod narrow with almost parallel sides and proximally enlarged, armed with an apical plumose seta. Pleopod 2 (Fig. 17D) exopod subovoidal with no apical plumose seta; endopod with first article nearly twice as long as second, a strong terminal bifid seta. Pleopods 3-5 exopods (Fig. 17E-G) ovoidal with a long plumose apical seta.

###### Etymology.

Latin: *sideralis* meaning of or with respect to the distant stars. The name refers to the international team of astronauts, taking part in training courses (ESA CAVES) in Sardinia organized by the European Space Agency, who first collected this new species.

###### Remarks.

The species occurs in subterranean freshwater ponds and streams in the karstic areas of Supramonte. It is closely related to the other two Sardinian stygobiotic species of *Alpioniscus* (*A.kuehni* and *A.stochi* sp. n.). It is readily distinguishable from *A.kuehni* by the smaller size (7.7 mm vs 12 mm), narrower body shape, shorter and more thickset antenna with smaller number of flagellar articles, presence on pereopod 7 ischium of a water conducting system, male pereopod 7 carpus shorter with more enlarged proximal part, male pleopod 1 exopod with narrower distal part, endopod shorter than exopod, male pleopod 2 exopod without setose apical seta. *Alpioniscussideralis* differs from *A.stochi* in its larger size (7.5 mm vs. 4.5 mm of the latter), antennula with a group of subapical aesthetascs, more numerous flagellar articles of the antenna (10-11 vs. 5-7), maxillipedal basis with a rounded instead of triangular lobe on outer margin, uropods with longer exopod and endopod, male pereopod 7 with thinner merus and carpus, merus with a distinct lobe on sternal margin.

#### Molecular results

After alignment, a 405 bp-long sequence dataset was obtained (see Table 1 for GenBank accession numbers). Since BI and ML analyses generated trees with identical topologies, only the BI tree is reported (Fig. 18 for more details). Each node is highly supported by both bootstrap values (BV) and posterior probability (PP). Among the species belonging to the genus *Alpioniscus*, A. (Alpioniscus) feneriensis from Piedmont is placed in the tree as the most external taxon. Its sister clade groups both *A.* (*Illyrionethes*) *strasseri* from Friuli Venezia Giulia and Sardinian species. All Sardinian species are clustered together in one clade. Sardinian terrestrial species (*Alpioniscusfragilis*, *A.thanit*, and *A.onnisi* sp. n.) set in a monophyletic group, showing a sister-taxon relationship with the clade including Sardinian aquatic species (*A.stochi* sp. n., *A.sideralis* sp. n., and *A.kuehni*). Within the terrestrial clade, *A.onnisi* sp. n. is the sister species of *A.thanit*. Within the aquatic clade, *A.kuehni* is the sister species of *A.sideralis* sp. n.

##### Key to species of *Alpioniscus* from Sardinia

**Table:** 

1	Body dorsal surface distinctly granulated (Fig. 1A)	**2**
–	Body dorsal surface smooth (Figs 5A, 9A, 12A, 15A)	**3**
2	Maximum length 14 mm; male pleopod 1 exopod triangular with outer margin almost straight (Fig. 3C); male pleopod 2 endopod with a subapical rounded lobe on distal article (Fig. 4A)	*** Alpioniscus fragilis ***
–	Maximum length 6-7 mm; male pleopod 1 exopod triangular with outer margin distinctly sinuous; male pleopod 2 endopod with no subapical rounded lobe on distal article	*** Alpioniscus thanit ***
3	Telson with distal part triangular (Fig. 5E); maxillipedal endite narrow with thickset apical penicil (Fig. 6E)	*** Alpioniscus onnisi ***
–	Telson with distal part trapezoidal (Figs 9E, 12E, 15E); maxillipedal endite enlarged, rectangular with small penicil near distal-medial corner (Fig. 10E, 13E, 16E)	**4**
4	Maximum length 15 mm; antenna thin and long, reaching pereonite 7 (Fig. 9A); antennal flagellum with 20-30 articles (Fig. 9G); pereopod 7 ischium with no trace of water conducting system (Fig. 11B)	*** Alpioniscus kuehni ***
–	Maximum length 7.5 mm; antenna thicker and much shorter than pereon (Figs 12A, 15A); antennal flagellum with up to 11 articles (Figs 12H, 15G); pereopod 7 ischium with distinct water conducting system (Figs 14B, 17B)	**5**
5	Maximum length 4.5 mm; antennula with two apical aesthetascs (Fig. 12G); antennal flagellum with 5 to 7 articles; male pereopod 7 merus with no lobe on sternal margin and thickset carpus (Fig. 14B); pleopod 3-5 exopods with no setose apical seta (Fig. 14E–F)	*** Alpioniscus stochi ***
–	Maximum length 7.5 mm; antennula with two apical and 3 subapical aesthetascs (Fig. 15F); antennal flagellum with 10 or 11 articles; male pereopod 7 merus with triangular lobe on sternal margin and slender carpus (Fig. 17B); pleopod 3-5 exopods with setose apical seta (Fig. 17E–F)	*** Alpioniscus sideralis ***

## Discussion

According to the data of the present study, the genus *Alpioniscus* is represented in Sardinia by six different species inhabiting various underground environments within a fragmented karst area of limited extension in the central-eastern and south-eastern part of the island (Fig. 19). All six species have morphological characters corresponding to the diagnosis of the subgenus Illyrionethes proposed by Verhoeff (1927), i.e., the distal article of the male pleopod 2 endopod shorter than or of equal length as the second article. This conclusion based on morphology is also supported by the molecular comparison with Alpioniscus (Illyrionethes) strasseri, type species of the subgenus Illyrionethes, and A. (Alpioniscus) feneriensis. However, a definite assessment of the taxonomic status within the genus *Alpioniscus* can be reached only after the comparison of all, or most of the species belonging to the genus from its entire distribution area.

According to the molecular phylogeny, the six Sardinian species are grouped into two distinct clades, one including the three terrestrial species (*A.fragilis*, *A.thanit*, and *A.onnisi*) and the other the three aquatic species (*A.stochi*, *A.sideralis*, *A.kuehni*). It seems most probable that the aquatic mode of life evolved once and then the aquatic species split into other species either by vicariant events related to the karst areas or by dispersal along subterranean aquifers with subsequent isolation. The dispersal in subterranean waters seems to be possible as demonstrated by the presence of *A.sideralis* and *A.kuehni* in two separated, even if close, karst areas (see Fig. 19). Dispersal of aquatic species across terrestrial habitats is unlikely. The presence of both terrestrial and aquatic species in the same genus is uncommon among Oniscidea. The only other case known so far is represented by the genus *Trogloniscus* Taiti & Xue, 2012, which includes three terrestrial and two aquatic species from caves in southern China (Taiti and Xue 2012).

*Alpioniscusfragilis* is a terrestrial species distributed along an area covering that of the whole genus in Sardinia. This species is mainly troglobiotic, but it can be found also in endogean habitats (e.g., under big stones near Lecorci Falls, Ogliastra) and in an aquatic environment (e.g., under submerged stones in Bue Marino Cave). So, the species shows a remarkable adaptive plasticity which allowed it to be distributed along a large and discontinuous area in suitable ecological conditions. All the other species have more restricted distributions. In some caves both terrestrial and aquatic species occur sympatrically. *Alpioniscusfragilis* coexists with two stygobiotic species, i.e., *A.kuehni* in the Grotta del Bue Marino, and *A.sideralis* in the Grotta Lovettecannas and Grotta Istirzili. In the Grotta Su Bentu *A.fragilis* occurs together with the two stygobiotic *A.kuehni* and *A.sideralis*, while in the Grotta Su Palu with the stygobiotic *A.stochi* and *A.sideralis*. The last two species share adaptive traits linked to the aquatic environment with *A.kuehni*, e.g., the quadrangular endite of the maxilliped, but also aspects that recall a previous existence in terrestrial environments, i.e., the water conducting system on the ischium of the pereopod 7. It is interesting to notice that some aquatic species co-occur in the same cave (i.e., *A.kuehni* and *A.sideralis* in the Grotta Su Bentu, and *A.stochi* and *A.sideralis* in the Grotta Su Palu), while this does not happen with the terrestrial species.

This richness in Sardinian *Alpioniscus* species is probably due to the complex geological and palaeoecological events that affected Sardinia, such as marine ingression or strong ecological variations that isolated the small karst islands (Zattin et al. 2008; Lichter et al. 2010), interrupting gene flow among populations and promoting speciation. Migratory events of underground fauna of the archipelago among the various geographical units may have occurred during the emersion periods with suitable environmental conditions. The co-occurrence of two aquatic species in the same cave may be due to subsequent invasions or different habitat preference. This last condition might be true for *A.stochi*, which occurs under stones on the bottom of the subterranean stream in the Grotta Su Palu, and *A.sideralis*, which is found on the submerged walls of the same stream. Two or more stygobiotic oniscidean species (genus *Haloniscus* Chilton, 1920) in the same subterranean aquifer are known also from some calcretes in Western Australian (Taiti and Humphreys 2001; Cooper et al. 2008).

## Supplementary Material

XML Treatment for Alpioniscus fragilis

XML Treatment for Alpioniscus thanit

XML Treatment for Alpioniscus onnisi

XML Treatment for Alpioniscus kuehni

XML Treatment for Alpioniscus stochi

XML Treatment for Alpioniscus sideralis
